# Norepinephrine in septic shock: catecholamine burden, immunometabolic reprogramming, and precision vasopressor therapy

**DOI:** 10.3389/fimmu.2026.1935801

**Published:** 2026-10-22

**Authors:** Xiaoxi Yan, Miaobo Li, Caifen Yan, Yihan Dang, Yating Lv, Tongqin Li, Jing Zhang, Yuge Wu, Li Ma

**Affiliations:** Department of Critical Care Medicine, The Second Hospital & Clinical Medical School, Lanzhou University, Lanzhou, Gansu, China

**Keywords:** high catecholamine burden, immunometabolism, immunoparalysis, neuroimmune regulation, norepinephrine, precision vasopressor therapy, septic shock, β2-adrenergic receptor

## Abstract

Septic shock is a life-threatening syndrome characterized by circulatory failure, immune dysregulation, neuroendocrine activation, and cellular metabolic dysfunction. Norepinephrine (NE) remains the first-line vasopressor for early resuscitation because it restores vascular tone, increases mean arterial pressure, and maintains vital organ perfusion. However, emerging evidence from neuroimmunology and immunometabolism suggests that NE also acts beyond hemodynamic support. This review reexamines the dual role of NE in septic shock from the perspective of high catecholamine burden, which integrates endogenous sympathetic activation, exogenous NE exposure, receptor signaling, and downstream immune-metabolic effects. Prolonged or high-dose NE exposure may regulate NF-κB activation, cytokine release, IL-10 production, phagocytic killing, antigen presentation, and lymphocyte effector function through adrenergic receptor-dependent pathways, particularly β2-adrenergic receptor/cAMP/PKA signaling. These effects may limit excessive inflammation during early disease but may also aggravate immunoparalysis in selected patients. NE may further influence glycolysis, mitochondrial oxidative phosphorylation, reactive oxygen species generation, and metabolic signaling pathways involving HIF-1α, AMPK, and mTOR, thereby reshaping immune-cell activation thresholds and antimicrobial capacity. Catecholamine signaling may also affect host-pathogen interactions by modulating bacterial growth, iron acquisition, and biofilm formation in specific experimental settings. Importantly, associations between NE exposure and adverse outcomes are strongly confounded by shock severity, infection burden, vascular hyporesponsiveness, and organ dysfunction. Future studies should integrate NE dose trajectories, cumulative exposure, immune phenotypes, metabolic states, and infection outcomes to clarify when catecholamine burden shifts from adaptive circulatory support to a potential driver of immune-metabolic dysfunction. This framework may support precision vasopressor strategies that balance hemodynamic load, immune function, and metabolic adaptability while preserving effective tissue perfusion.

## 1 Introduction

Septic shock is one of the most severe clinical phenotypes of sepsis. According to the Sepsis-3 definition, sepsis is life-threatening organ dysfunction caused by a dysregulated host response to infection. Septic shock represents a subset of sepsis with profound circulatory, cellular, and metabolic abnormalities. Clinically, it requires vasopressors to maintain a mean arterial pressure (MAP) of at least 65 mmHg after adequate fluid resuscitation, together with a serum lactate level greater than 2 mmol/L (1). This definition emphasizes that septic shock is not only a state of hypotension. It also involves tissue hypoperfusion, microcirculatory dysfunction, immune dysregulation, and cellular metabolic failure.

Sepsis remains a major global health burden. In 2017, approximately 48.9 million cases of sepsis and more than 11 million sepsis-related deaths were reported worldwide (2). Despite advances in antimicrobial therapy, resuscitation, and organ support, sepsis and septic shock are still associated with high mortality and long-term disability (3). Current treatment can restore circulation and support failing organs. However, effective strategies for correcting immune imbalance, microcirculatory dysfunction, and cellular metabolic abnormalities remain limited. This gap has shifted attention from simple circulatory rescue toward integrated restoration of vascular, immune, and metabolic homeostasis.

The understanding of sepsis pathophysiology has changed substantially. Early concepts mainly emphasized uncontrolled systemic inflammation. Pathogen-associated molecular patterns (PAMPs) and damage-associated molecular patterns (DAMPs) activate innate immune pathways. These signals induce inflammatory mediators such as tumor necrosis factor-α (TNF-α), interleukin-1β (IL-1β), and IL-6 (4). However, clinical trials targeting single cytokines or isolated inflammatory cascades have not consistently improved outcomes (5). These failures suggest that sepsis cannot be explained by excessive inflammation alone. It is a dynamic and heterogeneous disorder of the host response.

Increasing evidence shows that inflammation and immunosuppression can coexist early in sepsis. Their relative intensity varies across patients, organs, and disease stages (4). Decreased monocyte human leukocyte antigen-DR (mHLA-DR) expression, lymphocyte apoptosis, T-cell exhaustion, regulatory T-cell expansion, and sustained IL-10 elevation are typical features of sepsis-induced immunoparalysis (6). This immune phenotype is associated with secondary infection, opportunistic infection, viral reactivation, prolonged intensive care unit stay, and poor outcomes. Therefore, septic shock should be understood as a dynamic syndrome involving both immune activation and immune failure.

Circulatory dysfunction further interacts with immune and metabolic abnormalities. Septic shock is characterized by vasodilation, reduced vascular responsiveness, endothelial injury, glycocalyx degradation, microthrombus formation, and heterogeneous capillary perfusion. These changes may uncouple systemic hemodynamic improvement from tissue oxygen delivery (7). Mitochondrial dysfunction and impaired cellular oxygen utilization can also cause cytopathic hypoxia, even after macrocirculatory variables improve (8). Thus, achieving a MAP target does not necessarily indicate full recovery of tissue perfusion, immune function, or cellular metabolism.

Norepinephrine (NE) remains the first-line vasopressor for adults with septic shock. The latest Surviving Sepsis Campaign 2026 guidelines recommend NE as the first-line agent. When NE requirements increase and MAP remains difficult to maintain, vasopressin may be added to reduce further escalation of NE dose (9). The classic hemodynamic effect of NE mainly depends on α1-adrenergic receptor-mediated vasoconstriction. NE also supports cardiac output through β1-adrenergic effects (10). In the CENSER trial, early low-dose NE improved shock control by 6 hours, while a mortality benefit was not established (11). Therefore, NE remains essential for early circulatory support. Its immediate clinical value lies in restoring perfusion pressure and stabilizing systemic hemodynamics.

However, the biological significance of NE extends beyond vasoconstriction. NE is also a major effector molecule of the sympathetic nervous system. Multiple immune cells express adrenergic receptors, including monocytes, macrophages, neutrophils, dendritic cells, natural killer cells, T cells, and B cells. Among these receptors, the β2-adrenergic receptor (β2-AR) is particularly important in immune regulation (12). Through β2-AR/cyclic adenosine monophosphate (cAMP)/protein kinase A (PKA) signaling, NE can regulate nuclear factor-κB activation, cytokine release, IL-10 production, phagocytic killing, antigen presentation, and lymphocyte effector function (13). Experimental and translational studies further suggest that sustained NE exposure may impair anti-infective immune responses and promote bacterial dissemination in sepsis models (14). These findings create a clinically relevant tension. NE restores circulatory stability, but prolonged or high-dose exposure may amplify immunosuppression in selected patients.

This tension may have an immunometabolic basis. Immune-cell effector functions depend on specific metabolic programs. Pro-inflammatory macrophages and effector T cells usually rely on increased glycolysis. In contrast, immune exhaustion and impaired antigen presentation are often accompanied by mitochondrial dysfunction, abnormal oxidative phosphorylation, and metabolic hyporesponsiveness (15). In sepsis- and trauma-induced immune dysfunction, leukocyte metabolic remodeling, mitochondrial dysfunction, and altered redox homeostasis are important mechanisms of immune hyporesponsiveness (16). NE can also influence immune-cell mitochondrial metabolism and reactive oxygen species (ROS) signaling in experimental systems (17). These findings support NE as a regulator of immunometabolism alongside the broader metabolic disturbances caused by sepsis. Thus, NE-related immunosuppression may reflect more than transient cytokine changes. It may involve persistent remodeling of immune-cell metabolism and effector capacity.

Sepsis itself induces strong neuroendocrine activation. Infection, tissue injury, hypoperfusion, and inflammatory signals activate the sympathetic nervous system and the hypothalamic-pituitary-adrenal (HPA) axis. This promotes endogenous catecholamine and glucocorticoid release to maintain blood pressure, cardiac output, and organ perfusion (18). In early stress, this response is adaptive. During persistent infection, excessive sympathetic activation may lead to sustained endogenous catecholamine elevation. Exogenous NE infusion is then superimposed on an already activated adrenergic system (19). This state can be conceptualized as high catecholamine burden. It describes cumulative endogenous and exogenous catecholamine exposure, with receptor sensitivity and downstream signaling modifying the biological response. In addition, catecholaminergic drugs may influence bacterial growth, iron acquisition, and biofilm formation. This extends the potential role of NE to host-pathogen interactions (20).

NE therefore has a dual role in septic shock. It is indispensable for early hemodynamic rescue, but it may also shape immune function, metabolic programs, pathogen behavior, and organ recovery through adrenergic signaling (21). This review discusses how high catecholamine burden may connect NE exposure, immune dysregulation, immunometabolic reprogramming, and precision vasopressor therapy in septic shock. Clarifying these mechanisms may help explain the tension between vasopressor benefit and immune cost. It may also support strategies to reduce unnecessary catecholamine exposure, identify high-risk patient subgroups, and develop more individualized vasopressor therapy.

## 2 Immune dysregulation and high catecholamine burden in septic shock

NE is essential for circulatory resuscitation in septic shock. It rapidly restores MAP, improves systemic hemodynamic stability, and maintains perfusion of vital organs (9). However, NE is also a major effector molecule of the sympathetic nervous system. Through adrenergic receptors, it can influence immune-cell activity, host defense, and metabolic adaptation. Sepsis is not driven by a single inflammatory pathway. It is shaped by inflammation, immunosuppression, endothelial dysfunction, coagulation abnormalities, and metabolic failure (4). In this context, sustained endogenous and exogenous catecholamine signaling may link hemodynamic therapy with immune dysregulation and metabolic reprogramming.

### 2.1 Dynamic immune imbalance in sepsis

The immune response in sepsis is dynamic and heterogeneous. Early infection activates innate immune pathways through pathogen-associated and damage-associated signals. These signals promote pathogen recognition, immune-cell recruitment, and inflammatory mediator release (22). When the response becomes excessive or poorly controlled, it can contribute to endothelial injury, vascular leakage, coagulation activation, and microcirculatory dysfunction (7). Therefore, inflammation in sepsis is not uniformly protective or harmful. Its impact depends on timing, intensity, tissue context, and host reserve.

A purely inflammatory model cannot explain the complexity of sepsis. Clinical trials targeting single cytokines or isolated inflammatory cascades have not consistently improved outcomes (5). Current evidence supports a more complex model in which pro-inflammatory activation and immunosuppression may coexist from the early phase of disease (4). Their balance varies across patients, organs, pathogens, and disease stages. This heterogeneity helps explain why uniform immune-modulating strategies have been difficult to translate into clinical benefit.

Immunoparalysis is a clinically important phenotype of sepsis-induced immune dysfunction. It is characterized by impaired antigen presentation, reduced mHLA-DR expression, lymphopenia, T-cell exhaustion, immune checkpoint activation, and sustained anti-inflammatory signaling (6, 23). Persistent low mHLA-DR reflects monocyte hyporesponsiveness and is associated with infection risk and poor outcomes (24, 25). These markers characterize sepsis-associated immune dysfunction but have not been validated as measures of NE-specific effects. Adaptive immune dysfunction also contributes to this state. Lymphocyte apoptosis, impaired T-cell effector function, and immune checkpoint pathways can weaken pathogen control and increase susceptibility to secondary infection (26, 27).

Recent transcriptomic and multi-omics studies further show that sepsis contains distinct molecular and immune endotypes. These endotypes differ in inflammatory activity, immunosuppression, metabolic features, and mortality risk (28). This has direct relevance to NE. The immune effects of NE should not be described as simply anti-inflammatory or immunosuppressive. They are likely to depend on immune phenotype, disease stage, receptor expression, and metabolic state.

### 2.2 Formation of high catecholamine burden

Neuroendocrine activation is a core component of the host response in septic shock. Infection, tissue injury, hypoperfusion, pain, hypoxia, and inflammatory mediators activate the autonomic nervous system. These signals increase sympathetic output and promote the release of NE, epinephrine, dopamine, and other catecholamines from sympathetic nerve terminals and the adrenal medulla (29). In parallel, activation of the HPA axis increases glucocorticoid secretion and supports cardiovascular adaptation during acute stress (30).

In early shock, this stress response is adaptive. It supports blood pressure, cardiac output, and perfusion of vital organs. During persistent infection and severe inflammation, however, sympathetic activation may remain high for a prolonged period. Endogenous catecholamine levels can remain elevated, while exogenous NE infusion further increases adrenergic stimulation. In treated septic shock, endogenous sympathetic stress is therefore often superimposed on continuous vasopressor exposure.

This combined state can be described as high catecholamine burden. It does not refer only to a single peak hormone level or a single NE dose. It encompasses the intensity and duration of endogenous and exogenous catecholamine exposure. Receptor expression, sensitivity, and downstream signaling modify the biological response and may vary across immune cells and disease stages. This concept expands the clinical meaning of NE exposure. It links vasopressor therapy with immune-cell signaling, metabolic adaptation, and host-pathogen interactions. Here, the term denotes a conceptual framework rather than a validated score or threshold. Circulating concentrations reflect release, infusion, and clearance, whereas NE dose trajectories and cumulative doses describe administered exposure. Neither captures total tissue exposure. NE-equivalent doses summarize vasopressor support and may include non-catecholamine agents; they cannot quantify total catecholamine burden. Receptor-related biomarkers may characterize cellular responsiveness but require validation.

The exogenous component of high catecholamine burden is potentially modifiable. Traditional vasopressor therapy focuses on achieving a target MAP and maintaining organ perfusion. Recent studies on vasopressor selection and timing suggest that exposure intensity and non-hemodynamic effects should also be considered (31). Non-adrenergic vasopressors may reduce adrenergic drug exposure in patients with vasodilatory shock, although drug effects, timing, and patient selection remain heterogeneous (32). Vasopressin and related strategies are often discussed within decatecholaminization approaches because they may reduce the need for further NE escalation (33). Reduced NE requirements alone do not demonstrate immune or metabolic recovery. Catecholamine-sparing treatment must preserve adequate perfusion and hemodynamic stability.

### 2.3 High catecholamine burden as a link between immune remodeling and metabolic reprogramming

High catecholamine burden provides a useful framework for understanding the non-hemodynamic effects of NE. Catecholamines act on innate and adaptive immune cells through adrenergic receptors. Among these receptors, β2-AR is particularly important in immune regulation (12). Its downstream cAMP/PKA signaling can influence inflammatory transcription, cytokine release, phagocytic killing, antigen presentation, and lymphocyte responses (13).

These effects are context dependent. During early hyperinflammation, adrenergic anti-inflammatory signaling may help limit tissue injury. In an immunoparalyzed host, the same pathway could further impair antigen presentation and pathogen clearance. Stolk et al. demonstrated reduced pro-inflammatory mediator and ROS production and increased IL-10 *in vitro* and in LPS-challenged mice. Continuous NE infusion increased bacterial dissemination in the cecal ligation and puncture model. In patients with septic shock, higher NE infusion rates were associated with a more anti-inflammatory cytokine balance (14). These findings demonstrate immune modulation and impaired host defense in experimental models. The patient association may also reflect shock severity and treatment indication.

The immune effects of catecholamines are closely linked to metabolism. Effective immune responses require rapid metabolic adaptation. Pro-inflammatory macrophages and effector T cells often increase glycolysis, whereas immune exhaustion and impaired antigen presentation are associated with mitochondrial dysfunction, abnormal oxidative phosphorylation, redox imbalance, and metabolic hyporesponsiveness (15, 16). Sepsis-induced immunoparalysis is also associated with broad defects in leukocyte energy metabolism (34). Thus, immune failure in sepsis is not only a problem of cytokine release or cell number. It also reflects impaired energy supply, mitochondrial function, and redox control.

NE may participate in this immunometabolic shift. NE alters mitochondrial respiration and redox signaling in non-septic experimental T-cell systems, supporting a direct role in immune-cell metabolism (17). These effects should be distinguished from the general metabolic defects of sepsis described above. HIF-1α, AMPK, and mTOR remain candidate links between adrenergic signaling and metabolic adaptation in septic immune cells. Their effects are context dependent, and their involvement should not be presented as a single established NE-driven pathway. NADPH oxidase-derived antimicrobial ROS and mitochondrial ROS are distinct; reduced ROS output alone does not establish mitochondrial dysfunction. NE-related immunosuppression should therefore not be explained only by cytokine remodeling. It may also reflect persistent changes in immune-cell metabolism and effector fate.

This creates a central conceptual tension. NE is required to restore perfusion during circulatory failure. Yet high-dose or prolonged exposure may enhance adrenergic signaling in patients who already have immune dysregulation and high endogenous sympathetic stress. These effects provide a mechanistic basis for investigating whether NE exposure impairs pathogen clearance, delays immune recovery, or contributes to persistent infection in susceptible patients (13). Catecholaminergic drugs may also influence host-pathogen interactions by affecting bacterial growth, iron acquisition, and biofilm formation, although the clinical contribution of this mechanism remains uncertain (20). Thus, high catecholamine burden may help explain how a life-saving vasopressor can also carry potential immune and metabolic costs. The proposed framework linking septic shock, catecholamine exposure, immune dysregulation, immunometabolic reprogramming, and precision vasopressor therapy is summarized in **Figure 1**.

**Figure 1:**
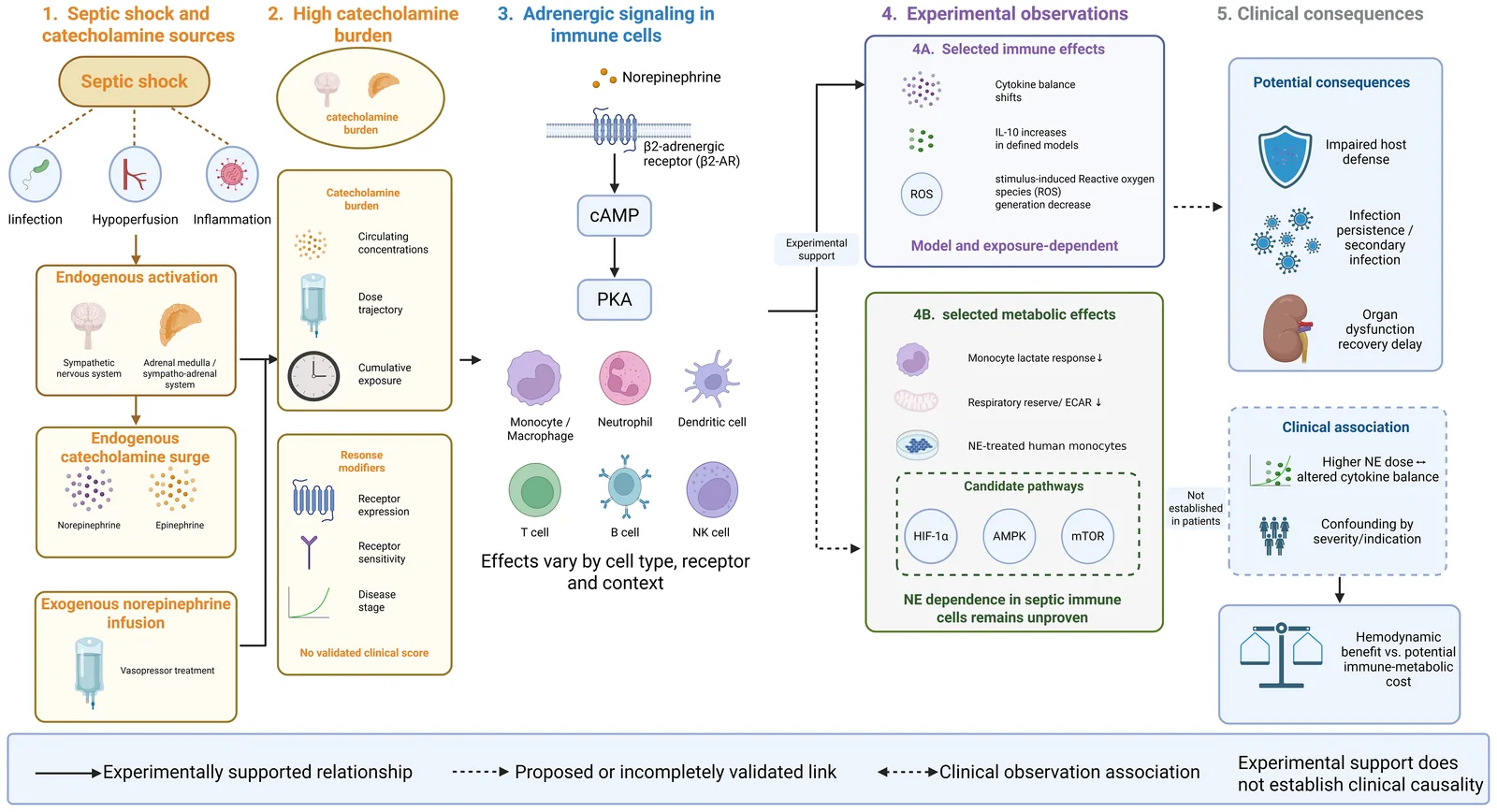
Catecholamine burden and norepinephrine-associated immune and metabolic changes in septic shock. Septic shock activates endogenous catecholamine release through sympathetic and sympathoadrenal pathways. Exogenous norepinephrine (NE) adds to this exposure. Catecholamine burden integrates circulating concentrations, dose trajectory, and cumulative exposure, with responses modified by receptor expression, receptor sensitivity, and disease stage. It represents a conceptual framework without a validated clinical score. The β_2_-AR/cAMP/PKA pathway illustrates an adrenergic mechanism underlying selected immune responses. Experimental findings include altered cytokine balance, increased IL-10 production, and reduced stimulus-induced ROS generation in defined models. In human monocyte experiments, NE attenuated LPS-induced lactate production and reduced spare respiratory capacity and maximal ECAR. These findings are model- and exposure-dependent. HIF-1α, AMPK, and mTOR are candidate metabolic regulators whose direct NE dependence in septic immune cells remains unconfirmed. Impaired host defense, persistent or secondary infection, and delayed organ recovery represent potential clinical consequences requiring further validation. Associations between NE dose and immune alterations may be confounded by disease severity and treatment indication. Solid arrows indicate experimentally supported relationships; dashed arrows indicate proposed or incompletely validated links; bidirectional dashed arrows indicate clinical observational associations. Potential immune-metabolic effects are considered alongside the established hemodynamic benefits of NE. β_2_-AR, β_2_-adrenergic receptor; cAMP, cyclic adenosine monophosphate; PKA, protein kinase A; IL-10, interleukin-10; ROS, reactive oxygen species; LPS, lipopolysaccharide; ECAR, extracellular acidification rate; HIF-1α, hypoxia-inducible factor 1-alpha; AMPK, AMP-activated protein kinase; mTOR, mechanistic target of rapamycin. Created in BioRender. Yan, Xiao. (2026) https://BioRender.com/a5iqc86.

## 3 Immunomodulatory mechanisms of norepinephrine

NE is not only a vasopressor. It is also a key mediator of sympathetic immune regulation. Sympathetic nerve fibers innervate lymphoid organs and inflamed tissues, and immune cells express adrenergic receptors. These features allow NE to participate directly in neuroimmune communication (35). During septic shock, endogenous sympathetic activation is already increased. Exogenous NE therapy further enhances adrenergic signaling. As a result, NE may regulate cytokine release, phagocytic killing, antigen presentation, and lymphocyte effector function (36, 37).

The immune effects of NE are cell specific and stage dependent. Moderate adrenergic signaling may limit excessive inflammation and reduce inflammatory tissue injury. Sustained catecholamine stimulation or prolonged exogenous NE exposure may have different consequences. It may suppress phagocytic killing, reduce antigen presentation, enhance anti-inflammatory mediator release, and impair lymphocyte effector function (13). Experimental sepsis studies show that NE can enhance anti-inflammatory responses, reduce some pro-inflammatory and oxidative killing responses, and compromise host defense (14). Therefore, NE should not be classified simply as protective or harmful. Its final effect depends on disease stage, cell type, receptor profile, exposure intensity, and local inflammatory context (18).

### 3.1 Adrenergic receptors and cell-specific immune responses

The molecular basis of NE-mediated immune regulation is adrenergic receptor expression on immune cells. Adrenergic receptors are G protein-coupled receptors and include α1, α2, β1, β2, and β3 subtypes. β2-AR is the most widely studied subtype in immune regulation. Monocytes, macrophages, neutrophils, dendritic cells, and natural killer cells express adrenergic receptors. T cells and B cells can also sense sympathetic signals, mainly through β2-AR (12, 38). However, receptor composition and functional outcomes differ across cell populations.

β2-AR usually activates adenylyl cyclase through Gs proteins. This increases cAMP production and activates PKA and related transcriptional regulators. The β2-AR/cAMP/PKA axis can regulate inflammatory gene transcription, cell migration, phagocytic function, and lymphocyte responses (39). In contrast, α1- and α2-adrenergic receptors have more cell-type-specific roles. Their effects involve inflammatory signaling in some myeloid cells, regulation of neurotransmitter release, and changes in local vascular and tissue environments (36). Thus, the immune effects of NE are shaped by receptor distribution, ligand concentration, exposure duration, and cell activation state.

β2-AR is also relevant to adaptive immunity. Its expression and function in T cells and B cells vary with activation status, differentiation stage, and tissue location (38). Studies across immune homeostasis, cancer immunology, and autoimmune disease indicate that β2-AR signaling can regulate immune-cell activation, proliferation, differentiation, and cytokine secretion (39). In sepsis, these effects may contribute to immune hyporesponsiveness, especially when adrenergic signaling is sustained.

### 3.2 Cytokine remodeling through the β2-AR/cAMP/PKA axis

The β2-AR/cAMP/PKA axis is a key pathway by which NE reshapes cytokine responses. After NE binds to β2-AR, intracellular cAMP increases. This signal affects inflammatory gene expression through PKA, cAMP response element-binding protein, and related transcriptional regulators. In monocytic cells, β-adrenergic stimulation can inhibit NF-κB activation and reduce Toll-like receptor-induced pro-inflammatory responses (40). As a result, TNF-α, IL-1β, IL-6, and related mediators may decrease.

This effect may be beneficial during early hyperinflammation because it can limit inflammatory tissue injury. When infection persists or host immunity is already suppressed, however, excessive suppression of pro-inflammatory responses may reduce pathogen clearance. NE-mediated cytokine remodeling is also not limited to reduced pro-inflammatory cytokines. β2-AR activation can promote IL-10 production. IL-10 limits inflammatory injury, but sustained IL-10 elevation can suppress monocyte and macrophage activation, reduce antigen-presenting capacity, and maintain an immunosuppressive microenvironment (41).

This pathway is clinically relevant in septic shock. The experimental and patient findings described in Section 2.3 link NE exposure to anti-inflammatory cytokine remodeling (14). The balance between limiting tissue injury and impairing host defense depends on immune phenotype and inflammatory context. The β2-AR/cAMP/PKA-mediated signaling network and its major cell-specific immunomodulatory effects in septic shock are summarized in **Figure 2**.

**Figure 2:**
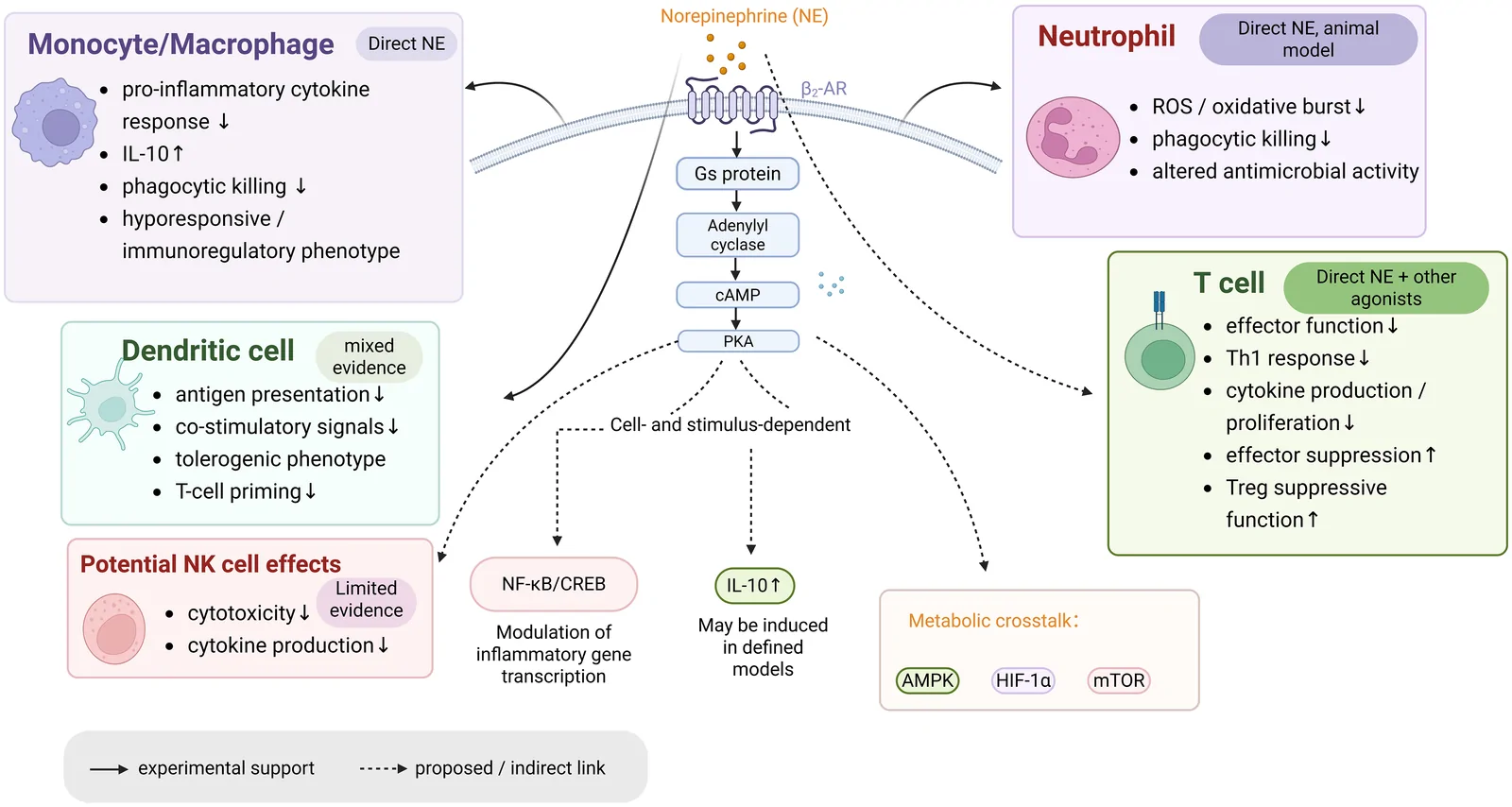
β_2_-Adrenergic signaling and immune-cell responses relevant to norepinephrine exposure in septic shock. Norepinephrine (NE) activates the β_2_-adrenergic receptor (β_2_-AR)/Gs/adenylyl cyclase/cAMP/protein kinase A (PKA) pathway. Downstream responses include modulation of inflammatory gene transcription and increased interleukin-10 (IL-10) production in defined experimental models. The cell panels summarize selected immunomodulatory responses involving monocytes/macrophages, neutrophils, dendritic cells, and T cells. Evidence derives from direct NE exposure and related adrenergic agonist studies *in vitro* and in animal models; enhanced regulatory T-cell function includes evidence from β_2_-AR agonist experiments. Impaired phagocytic killing and broadly reduced dendritic-cell antigen presentation, co-stimulation, or T-cell priming represent proposed functional consequences requiring model-specific confirmation. Natural killer-cell effects remain less well characterized in septic shock. AMPK, HIF-1α, and mTOR represent candidate metabolic links rather than a fixed downstream cascade. Solid arrows indicate experimentally supported signaling relationships or cell-level effects; dashed arrows indicate proposed or indirect links. Individual outcomes vary with cell type, stimulus, and exposure conditions and do not imply exclusive PKA dependence or established effects during clinical NE treatment. Gs, stimulatory G protein; cAMP, cyclic adenosine monophosphate; NF-κB, nuclear factor-kappa B; CREB, cAMP response element-binding protein; ROS, reactive oxygen species; Th1, type 1 helper T cell; Treg, regulatory T cell; NK, natural killer; AMPK, AMP-activated protein kinase; HIF-1α, hypoxia-inducible factor 1-alpha; mTOR, mechanistic target of rapamycin. Created in BioRender. Yan, Xiao. (2026) https://BioRender.com/4zo79cx.

### 3.3 Effects on innate immune cells

Monocytes and macrophages are central to innate immunity and sepsis-induced immunoparalysis. NE can affect their cytokine release, phagocytic killing, and antigen presentation through β2-AR/cAMP/PKA signaling. In cultured macrophages, β-adrenergic stimulation can shift profiles toward an M2-like immunoregulatory state and suppress some M1-related inflammatory programs (42). This shift may reduce tissue injury during early inflammation. In persistent infection or immunoparalysis, it may weaken pathogen clearance and delay immune recovery.

Neutrophils are essential for early pathogen control. Their antimicrobial activity depends on chemotaxis, adhesion, phagocytosis, degranulation, and oxidative burst. Adrenergic signaling can affect neutrophil migration, adhesion molecule expression, and oxidative killing (43). NE reduced ROS production in stimulated leukocytes, and increased bacterial dissemination was observed in a separate sepsis model (14). Neutrophil extracellular traps can restrict pathogens, but they can also promote thromboinflammation and endothelial injury (44). Direct evidence that NE regulates neutrophil extracellular traps in sepsis remains limited.

Dendritic cells link innate and adaptive immunity. Their migration, maturation, and antigen-presenting capacity determine T-cell priming. NE can regulate dendritic-cell migration and functional status through adrenergic receptors (45). β2-AR activation can enhance anti-inflammatory properties of dendritic cells and may favor tolerogenic immune responses (46). In septic shock, this shift may contribute to impaired antigen presentation and adaptive immune hyporesponsiveness.

Natural killer cells also express adrenergic receptors. Sympathetic activation is generally associated with reduced natural killer cell cytotoxicity and cytokine production (12). In sepsis, this may affect early antimicrobial defense and viral control. However, direct evidence for NE-mediated natural killer cell dysfunction in septic shock remains limited. This mechanism should be viewed as a possible component of catecholamine-related immune suppression, not as an established therapeutic target.

### 3.4 Effects on adaptive immunity and immune checkpoint pathways

Adaptive immune dysfunction is a major component of sepsis-induced immunoparalysis. Reduced T-cell numbers, impaired effector function, and T-cell exhaustion are especially important. β2-AR signaling can act directly on T cells and suppress T-cell receptor-mediated proliferation, cytokine production, and effector function (38). Estrada et al. showed that sympathetic signaling suppresses CD8+ T-cell effector function through β2-AR (47). In sepsis, this effect may weaken control of viral reactivation, secondary infections, and intracellular pathogens.

NE can also regulate CD4+ T-cell subsets. β2-AR signaling can affect the balance between type 1 and type 2 helper T-cell responses. It can also enhance the suppressive function of Foxp3+ regulatory T cells through a PKA-dependent mechanism (48). During hyperinflammation, this regulation may reduce immune-mediated tissue injury. During immunoparalysis, it may further suppress effector T-cell responses and delay immune recovery.

B cells also express β2-AR. Sympathetic signaling can affect antibody production, cytokine secretion, and interactions between B cells and T cells (38). The role of B cells in NE-related immunosuppression during sepsis remains less clear. It may contribute to long-term immune dysfunction, but current evidence is insufficient to define it as a major independent mechanism.

Immune checkpoint pathways are important in sepsis-induced adaptive immunosuppression. The PD-1/PD-L1 pathway contributes to T-cell exhaustion, myeloid-cell dysfunction, and impaired pathogen clearance (27). At present, NE should not be assumed to directly drive PD-1/PD-L1 upregulation in patients with sepsis. However, sustained adrenergic signaling, T-cell exhaustion, and immune checkpoint activation may interact. Future studies should examine NE exposure, immune phenotype, and checkpoint dynamics together.

### 3.5 Stage-dependent dual effects of NE on immunity

Current evidence supports a model shaped by disease stage and baseline immune phenotype, rather than a fixed early-to-late switch. In hyperinflammatory settings, NE may limit excessive inflammation through β2-AR/cAMP/PKA signaling. It can reduce pro-inflammatory mediator release and enhance IL-10 responses. These effects may reduce inflammatory tissue injury. However, when catecholamine stimulation persists, or when immunoparalysis has developed, the same pathway may become detrimental. It may suppress antimicrobial functions in monocytes, macrophages, and neutrophils. It may also impair dendritic-cell antigen presentation, reduce T-cell effector responses, and promote tolerogenic immune phenotypes (21).

This stage dependence explains why the clinical immune effects of NE are difficult to interpret. NE is essential for hemodynamic rescue, but its immunologic consequences depend on baseline immune status, endogenous catecholamine tone, exogenous dose, exposure duration, and receptor signaling intensity (19). The relevant clinical question is therefore not simply whether NE is used. It is how much adrenergic signaling the patient receives, for how long, and in which immune phenotype.

Sustained adrenergic stimulation may also shape longer-term immune remodeling. In a non-septic model, human monocytes were exposed to NE or epinephrine for 24 hours and then rested and differentiated for five days. Seahorse analysis showed increased glycolysis and oxidative phosphorylation. LPS restimulation elicited greater TNF-α production. This suggests that high catecholamine environments may influence immune-cell fate beyond short-term signal transduction (49). These observations support a broader view of NE in septic shock. NE may act not only as a circulatory support drug but also as a regulator of immune-cell function and recovery.

Together, these findings suggest that norepinephrine does not exert a uniform immunosuppressive effect across all leukocyte populations. Instead, its immune consequences depend on cell type, activation state, receptor sensitivity, disease stage, and cumulative adrenergic exposure. The major cell-specific immune effects of norepinephrine and their potential precision-monitoring relevance are summarized in **Table 1**.

**Table 1:** Cell-specific immune effects of norepinephrine in septic shock.

Immune-cell type	Functional domain	Signaling node	Reported finding	Evidence/experimental setting	Potential assessment readouts	Ref.
Monocytes/macrophages	Cytokine regulation	β2-AR/cAMP/PKA	TNF-α ↓; IL-10 ↑	Human monocytes *in vitro*; direct NE exposure	TNF-α/IL-10 response	(14)
Monocytes	Energy metabolism and oxidative burst	Not specified	Glycolysis and OXPHOS ↓; stimulated ROS ↓	Human monocytes *in vitro*; NE; separate metabolic and ROS assays	ECAR/OCR; ROS burst	(14)
Macrophages	Polarization	β2-AR-related transcription	M2-like gene profile ↑; selected M1-related programs ↓	Murine macrophages *in vitro*; isoproterenol	Polarization markers; cytokine profile	(42)
Monocytes/macrophages	Antigen presentation	MHC II/mHLA-DR (markers)	Low mHLA-DR marks sepsis-related immune dysfunction; NE-specific causation unresolved	Clinical sepsis immunophenotyping; general sepsis evidence	mHLA-DR expression	(25)
Monocytes	Trained immunity	β-AR/cAMP	Glycolysis and OXPHOS ↑; restimulated TNF-α ↑	Human monocytes *in vitro*; NE/epinephrine for 24 h, then five days of rest and differentiation	ECAR/OCR; restimulation response	(49)
Neutrophils	Trafficking	adrenergic regulation	Chemotaxis and adhesion altered	Reviewed *in vitro*/preclinical studies; NE or adrenergic agonists	Chemotaxis; CD11b/CD62L	(12, 43, 102)
Neutrophils	Phagocytosis	adrenergic regulation	Phagocytosis ↓ in selected exposure settings	Reviewed non-septic *in vitro* studies; NE effects depend on dose and model	Phagocytosis assay	(12, 43)
Neutrophils	Oxidative burst	Not specified	Stimulated ROS production ↓	LPS-challenged mice; NE infusion	Oxidative burst capacity	(14)
Dendritic cells	Migration	α1b-AR	Immature DC migration ↑	Murine DC cultures/skin explants; direct NE exposure	DC migration assay	(45)
Dendritic cells	Antigen handling and T-cell polarization	β2-AR (cytokines/Treg); α-AR (uptake, suggested)	Epinephrine: uptake ↑; β2-AR: IL-10 and Treg induction ↑, IL-12 ↓; MHC II/CD80/CD86 unchanged	Murine bone marrow-derived DCs *in vitro*; epinephrine/salbutamol	DC cytokines; antigen uptake; T-cell assays	(46)
CD4+ T cells	Cytokine responses and proliferation	β2-AR	NE: cytokine responses vary with activation state; fenoterol: proliferation ↓	Murine T-cell cultures; NE or fenoterol; NE findings reviewed	IFN-γ; IL-2; proliferation	(38, 48)
CD8+ T cells	Effector function	β2-AR/TCR	IFN-γ and TNF-α ↓; cytotoxicity ↓	Human and murine T cells *in vitro*; direct NE exposure	Cytokine and cytotoxicity assays	(47)
CD8+ T cells	Metabolic activation	β2-AR	GLUT1, glycolysis and mitochondrial respiration ↓	Murine T cells *in vitro*; isoproterenol during activation	GLUT1; ECAR/OCR	(55)
Regulatory T cells	Suppressive function	β2-AR/cAMP/PKA	Treg suppressive activity ↑	Murine T cells *in vitro*; fenoterol	Treg suppression assay	(48)
B cells	Humoral response	β2-AR	Antibody output altered; IgG1 ↑ in selected activation settings	Reviewed murine B-cell activation studies; NE or β2-AR agonists	Immunoglobulin output; B-cell subsets	(38, 103)
B cells	Trafficking	β2-AR/CCR7	Lymph-node egress ↓; circulating B cells ↓	Mouse models; selective β2-AR agonists	Circulating B-cell subsets	(104)
Natural killer cells	Cytotoxicity	β-adrenergic regulation	Cytotoxic activity ↓ *in vitro*	Human NK-cell assays with NE; reviewed experimental evidence	NK cytotoxicity assay	(12, 102, 103)

↑, increased; ↓, decreased, relative to the relevant control or comparator.

## 4 Norepinephrine-mediated immunometabolic reprogramming

Immune-cell effector functions depend on precise metabolic regulation. Cytokines, pattern recognition receptors, and transcription factors determine the direction of immune responses. Energy supply, substrate utilization, and redox status determine whether immune cells can migrate, phagocytose, kill pathogens, present antigens, and secrete cytokines (50). In sepsis, immunoparalysis is not only reflected by reduced cytokine release or decreased immune-cell numbers. It is also associated with higher activation thresholds, insufficient metabolic adaptation, and impaired effector function (34).

Pro-inflammatory monocytes, macrophages, and effector T cells usually increase glycolysis rapidly. This provides energy and metabolic intermediates required for inflammatory mediator synthesis, clonal expansion, and antimicrobial responses (51). Immune tolerance and exhaustion can involve altered glycolysis, mitochondrial respiration, and lipid metabolism, but the direction and functional significance of these changes vary with cell type and activation state (52). High catecholamine burden in septic shock may contribute to this transition. Direct NE-exposure experiments demonstrate changes in monocyte glycolytic and respiratory capacity and leukocyte oxidative responses, extending its immune effects beyond cytokine regulation (14). β2-AR/cAMP/PKA signaling provides a mechanistic basis for adrenergic immune regulation, although its contribution must be established separately for each metabolic endpoint. These findings support NE-associated immunometabolic remodeling as a potential contributor to immune hyporesponsiveness during septic shock. Key experimental and clinical studies on norepinephrine-related immune and immunometabolic effects, including exposure conditions, measured outcomes, and major limitations, are summarized in **Supplementary Table S1**.

### 4.1 Glycolytic hyporesponsiveness and impaired antimicrobial effects

Glycolysis is an important metabolic basis for acute antimicrobial immunity. During early infection, pathogen-associated molecular patterns and inflammatory cytokines can enhance glucose uptake in monocytes, macrophages, neutrophils, and effector T cells. They also increase the activity of key enzymes such as hexokinase, phosphofructokinase, and lactate dehydrogenase. Even when oxygen is sufficient, immune cells can preferentially rely on glycolysis to rapidly generate ATP and metabolic intermediates. This metabolic switch supports inflammatory gene transcription, phagocytic killing, cell migration, and clonal expansion (50). In LPS-activated macrophages, increased glycolysis is closely associated with HIF-1α activity and IL-1β induction. This suggests that glycolysis is not only an energy-producing process but also an important regulatory component of inflammation (53).

As sepsis progresses, increased glycolysis does not necessarily persist. In some patients, leukocytes fail to effectively initiate glycolysis after repeated infectious stimulation. Experimental endotoxin tolerance and leukocytes from patients with sepsis-associated immunoparalysis show impaired glycolytic and oxidative responses alongside reduced inflammatory responsiveness (34). These findings establish the sepsis-associated metabolic context in which NE acts; infection, hypoxia, and nutrient availability may also contribute. In this state, immune cells may remain viable but fail to mount an effective antimicrobial response. Recent reviews of metabolic changes in sepsis also show that abnormalities in glucose metabolism, amino acid metabolism, lipid metabolism, and mitochondrial function jointly shape immune-cell functional states at different disease stages (54).

NE may aggravate glycolytic hyporesponsiveness. Stolk et al. found reduced LPS-induced lactate accumulation in primary human monocytes after NE pretreatment (1 μM for 1 hour), followed by LPS stimulation (10 ng/mL for 24 hours). Separate live-cell assays with NE at 1 μM showed reduced spare respiratory capacity and maximal extracellular acidification. PKA inhibition partly reversed the lactate effect, supporting signaling-dependent regulation of glycolytic responsiveness (14). These metabolic measurements provide direct cellular evidence, while bacterial dissemination was assessed in a separate mouse model; metabolic mediation of the host-defense defect remains to be demonstrated. At the T-cell level, Qiao et al. treated anti-CD3/CD28-activated murine CD8+ T cells with isoproterenol (10 μM). GLUT1 expression and glucose uptake were assessed at 24–48 hours, and glycolysis and mitochondrial respiration after 48 hours. The inhibitory response was lost in β2-AR-deficient cells, supporting receptor-dependent restriction of activation-associated metabolism (55). This strengthens the adrenergic mechanism, while the synthetic agonist and nonseptic model distinguish it from direct NE exposure in septic shock. Neutrophils depend heavily on glycolysis for ATP, with the pentose phosphate pathway supplying NADPH for oxidative burst. Macrophages adjust both glycolytic and oxidative metabolism according to their activation state (50, 51). Dendritic-cell metabolism also changes during maturation, although direct NE-specific metabolic evidence is less developed than the monocyte data (50). In T cells, restricted glycolysis can impair proliferation, cytokine production, and effector differentiation. Together, these findings support a model in which high catecholamine burden can restrict metabolic adaptability in susceptible immune-cell populations. The response is shaped by receptor subtype, ligand concentration, exposure duration, inflammatory stimulus, and baseline immune state, rather than a uniform transition to metabolic suppression. Culture concentrations cannot be equated directly with clinical infusion rates.

The effect of NE on glycolysis remains context dependent. During excessive inflammatory activation, restraint of inflammatory glycolysis may reduce tissue injury. However, during persistent infection, immunoparalysis, or increased risk of secondary infection, insufficient glycolysis can limit host defense. In LPS-stimulated macrophages, IL-10 reduced glucose uptake and glycolysis while promoting oxidative phosphorylation and mitophagy through DDIT4-dependent mTOR inhibition (56). Together with direct evidence that NE can induce β2-AR-dependent IL-10 release (41), this provides a plausible indirect route linking adrenergic signaling to metabolic remodeling. The two mechanisms were demonstrated in different experimental settings, and their combined NE dependence requires testing. This response represents selective metabolic regulation rather than global energy failure.

### 4.2 Mitochondrial bioenergetic dysfunction and immune-cell hyporesponsiveness

Mitochondria are not only responsible for oxidative phosphorylation and ATP production. They also regulate ROS generation, inflammasome activation, apoptosis, metabolic intermediate supply, and mitochondrial DNA release (57). During sepsis, mitochondrial injury can occur in both immune cells and parenchymal cells. It is characterized by impaired electron transport chain function, reduced membrane potential, insufficient ATP production, abnormal mitochondrial dynamics, and disturbed quality control (57). These changes reduce the ability of cells to rapidly mobilize energy and execute effector functions after infectious stimulation.

Mitochondrial dysfunction in immune cells is closely related to immunoparalysis. Reduced glycolytic and oxidative metabolic responses have been documented in leukocytes from patients with sepsis-associated immunoparalysis (34). Defects in mitochondrial function can accompany impaired immune responses, but their respiratory phenotype varies across cell subsets and activation states. Therefore, mitochondrial dysfunction is not only a consequence of organ failure. It may also directly maintain immune hyporesponsiveness (58).

The monocyte findings described in Section 4.1 identify respiratory reserve as a direct NE-sensitive metabolic endpoint (14). A reduction in spare respiratory capacity indicates limited capacity to meet additional demand; it does not by itself establish structural mitochondrial injury, reduced ATP content, or the same defect across all leukocytes. Stimulated ROS production is a separate endpoint, whose source and contribution to microbial killing require distinct measurements. If NE-associated loss of respiratory reserve persists in septic shock, it could restrict the capacity to meet antimicrobial energy demands even after circulatory perfusion improves. Improvement in MAP does not necessarily indicate recovery of immune-cell energy metabolism. This provides a testable explanation for incomplete recovery of host defense after hemodynamic stabilization. Paired respiratory, viability, and microbial-killing measurements are needed to determine whether restoring metabolic reserve rescues antimicrobial function during NE exposure.

Mitochondrial quality control also deserves attention. Mitochondrial biogenesis, fusion and fission, and mitophagy jointly maintain the stability of the mitochondrial network. Severe infection can involve mitochondrial dysfunction and abnormal mitophagy. Insufficient clearance of damaged mitochondria can sustain inflammatory signaling and organ injury (59). Current evidence for direct NE regulation of mitophagy remains limited. However, persistent inflammation, energy stress, and adrenergic signaling may together affect damaged mitochondrial clearance and mitochondrial renewal. In patients with prolonged septic shock, impaired mitochondrial quality control may contribute to persistent inflammation, immunosuppression, and catabolic states.

### 4.3 ROS regulation and antimicrobial oxidative burst

ROS have dual roles in sepsis. Excessive ROS can cause lipid peroxidation, protein injury, DNA damage, endothelial barrier disruption, and organ dysfunction. However, appropriate ROS generation is essential for neutrophils and macrophages to kill pathogens (60). Neutrophil antimicrobial oxidative burst is largely generated by NADPH oxidase, particularly NOX2, and supports pathogen killing and some forms of NET formation. This is distinct from mitochondrial ROS involved in signaling and oxidative stress. Oxygen consumption during an oxidative burst can therefore reflect NADPH oxidase activity rather than mitochondrial oxidative phosphorylation (60). Immune cells therefore do not simply need reduced ROS. They need a balance between antimicrobial oxidation and oxidative injury.

Impaired stimulus-induced oxidative burst can coexist with increased basal or mitochondrial oxidative stress in sepsis. If monocytes, macrophages, and neutrophils cannot effectively initiate oxidative burst, pathogen clearance may be insufficient even when cell numbers are not markedly decreased. Adrenergic signaling can alter cellular redox responses, but a total ROS assay cannot determine whether NADPH oxidase-derived or mitochondrial ROS are affected (17). Stolk et al. reported reduced stimulated ROS responses in human monocytes and altered neutrophil oxidative responses in mouse endotoxemia. In a separate polymicrobial sepsis experiment, continuous NE infusion (3 μg/kg/min for 48 hours) increased spleen and liver bacterial counts; the blood increase was a nonsignificant trend (P = 0.06) (14). These findings link NE exposure to redox changes and compromised experimental host defense, without identifying ROS suppression as the causal mediator. Thoppil et al. also observed attenuation of stimulus-induced total ROS fluorescence, most consistently in CD3+ cells, after NE treatment of control-donor PBMCs (61). The reported 5 mM concentration and 16-hour stimulation limit extrapolation to clinical NE exposure. This study measures redox modulation rather than mitochondrial respiration or bacterial killing; reduced fluorescence could indicate lower oxidative stress as well as altered oxidative responsiveness.

ROS also participate in NET formation. NETs can restrict pathogen dissemination through DNA networks, histones, and granule proteins. However, excessive NET formation can promote thromboinflammation, endothelial injury, and microcirculatory dysfunction (44). The effect of NE on NETs may differ by disease stage. Insufficient ROS may weaken pathogen restriction. In contrast, uncontrolled NET formation may aggravate intravascular inflammation and organ injury. Current evidence for direct NE regulation of NETs remains limited. Future studies should distinguish infection stage, cell status, and pathogen burden.

Glycolysis, mitochondrial respiration, and oxidative burst jointly determine the redox state of immune cells. The NE–immunometabolic framework proposes that restricted metabolic adaptability and impaired antimicrobial oxidative responses may converge in susceptible cell populations. Their simultaneous occurrence and causal contribution to poor pathogen control have not yet been established in septic patients. Testing this metabolic-effector connection requires source-specific ROS assays and demonstration that metabolic or redox rescue restores microbial killing.

### 4.4 HIF-1α, AMPK, and mTOR signaling networks

Immunometabolic reprogramming is not only reflected by changes in metabolic flux. It also depends on the resetting of metabolic sensing pathways. HIF-1α, AMPK, and mTOR sense oxygen tension, energy status, and nutrient signals, respectively. Together, they regulate the transition of immune cells among inflammatory activation, metabolic adaptation, and functional suppression (50).

HIF-1α is an important transcription factor that promotes glycolysis and inflammation. Hypoxia, infection, and inflammatory stimulation can stabilize HIF-1α, upregulate glucose transport and glycolysis-related genes, and promote the production of inflammatory mediators such as IL-1β (62). During early infection, HIF-1α-mediated glycolytic enhancement supports antimicrobial immunity. However, sustained activation may also worsen inflammatory injury. HIF-1α is a candidate link between adrenergic signaling and glycolytic responsiveness because of its established role in inflammatory metabolic programming. A proposed mechanism is that NE-related signaling or secondary cytokine responses alter HIF-1α-dependent gene expression. Direct NE regulation of HIF-1α stability or activity has not been established in the septic immune-cell studies discussed here (17). This connection therefore requires cellular mechanistic validation, in addition to clinical evaluation.

AMPK is a major sensor of cellular energy stress. When ATP consumption increases or energy supply becomes insufficient, AMPK is activated. This promotes energy conservation, mitochondrial homeostasis, and autophagy regulation (63). AMPK activation often helps limit excessive inflammation and maintain cell survival. Its functional consequences should be interpreted by cell type and inflammatory context. Huang et al. found that AMPK deficiency increased PKM2-dependent glycolysis and HMGB1 release in monocytes/macrophages, whereas pharmacologic AMPK activation protected mice against experimental sepsis (64). Thus, reduced inflammatory glycolysis can coexist with protection rather than immune failure. NE-related metabolic stress could engage AMPK-dependent adaptation, but a direct NE-to-AMPK mechanism and its antimicrobial consequences remain to be tested. The central question is whether AMPK preserves cellular fitness or limits a particular effector response under the relevant exposure conditions.

mTOR regulates cell growth, protein synthesis, nutrient sensing, and immune-cell activation (65). mTOR activity is closely related to T-cell proliferation, effector differentiation, macrophage inflammatory responses, and metabolic switching. The consequences of mTOR activation or inhibition vary with cell differentiation, activation state, and mTOR complex, so neither direction denotes immune suppression uniformly (65, 66). For NE, the IL-10–DDIT4–mTOR mechanism described in Section 4.1 provides a plausible indirect connection between cytokine regulation and macrophage metabolic remodeling (41, 56). This mechanism can restrain inflammatory glycolysis while preserving mitochondrial quality. Direct regulation of mTOR by β2-AR/cAMP/PKA in septic immune cells remains to be tested separately.

HIF-1α, AMPK, and mTOR are not isolated pathways. Together, they determine the metabolic direction of immune cells. Short-term adrenergic signaling may help the host adapt to acute stress and limit inflammatory injury. Under sustained high catecholamine burden, altered regulation of these nodes could constrain metabolic flexibility and immune recovery. They form an interconnected regulatory network, rather than a demonstrated linear NE–HIF-1α–AMPK–mTOR pathway. Receptor blockade and pathway-specific perturbation, paired with metabolic flux and antimicrobial assays, would identify which connections mediate the NE response and which reflect infection, hypoxia, or secondary cytokine signaling.

### 4.5 Metabolic remodeling and sepsis-induced immunoparalysis

The NE–immunometabolic framework integrates direct NE-sensitive cellular endpoints with broader sepsis mechanisms and candidate signaling connections. Glycolytic hyporesponsiveness limits cytokine production and effector-cell activation. Mitochondrial dysfunction weakens energy reserve and stress responses. Restricted NADPH oxidase-dependent oxidative burst can compromise pathogen killing, whereas reduced mitochondrial or total ROS may instead reflect less oxidative stress. HIF-1α, AMPK, and mTOR offer candidate explanations for changes in metabolic adaptation and activation thresholds. Their overlap with sepsis-induced immunoparalysis makes the framework biologically coherent, while evidence from different models should not be treated as one established NE-dependent causal sequence (58).

This framework raises a clinically relevant question about the recovery of host defense after circulatory stabilization. NE rapidly restores perfusion pressure and provides short-term life support. Experimental findings raise concern that high-dose or prolonged exposure may compound host-defense defects in patients with immune hyporesponsiveness (21). The size and independence of this effect in clinical septic shock remain to be determined. Blood pressure improvement does not equal immune recovery. Some patients continue to show monocyte hyporesponsiveness, lymphocyte exhaustion, reduced neutrophil killing capacity, and increased secondary infection risk after circulatory indices stabilize. Immunometabolic dysfunction provides an important clue for explaining the dissociation between hemodynamic improvement and poor infection control.

Clinical metabolomics studies further show that patients with sepsis and septic shock have broad metabolic disturbances. Early clinical-metabolomic models showed that metabolic profiles can improve prediction of mortality risk in sepsis (67). A multicenter cohort study associated increased plasma acetylcarnitine with organ dysfunction and mortality risk, supporting its prognostic relevance in sepsis (68). This systemic metabolite measurement does not identify defective fatty acid oxidation in a particular immune-cell population. Integrated metabolomic and proteomic studies also showed marked abnormalities in amino acid metabolism and inflammation-related pathways in patients with sepsis (69). These studies define systemic sepsis-associated metabolic abnormalities, providing clinical context for NE-focused research rather than an NE-specific metabolic signature. Plasma metabolites reflect multiple tissues, organ clearance, nutrition, and treatment. Linking them to NE-associated immune-cell remodeling requires concurrent exposure measurements and cell-specific functional assays.

The experimental metabolic effects of NE support evaluating exposure intensity and duration alongside hemodynamic benefit in translational research. Current vasopressor decisions must prioritize adequate perfusion; incorporation of immune phenotype into NE selection or dose adjustment requires prospective validation. A more reasonable strategy is not to avoid NE. Instead, it is to reduce unnecessary high-dose exposure while ensuring tissue perfusion. Serial mHLA-DR, lymphocyte counts, IL-10, leukocyte metabolic assays, and mitochondrial measurements can characterize candidate research phenotypes. They indicate general immune or metabolic abnormalities and are not validated markers of NE-specific injury or thresholds for adjusting vasopressors. Lactate kinetics reflect systemic production and clearance and should not substitute for leukocyte glycolytic flux. A decrease in NE dose must be evaluated separately from immune recovery. Metformin is proposed for evaluation in a safety and feasibility trial protocol for sepsis-associated AKI (70). This supports investigation of metabolic intervention but provides no efficacy evidence for reversing NE-associated immune dysfunction. AMPK-directed or other metabolic treatments remain investigational in septic shock.

Therefore, NE is not only a circulatory support drug in septic shock. It may also alter the metabolic fate of immune cells. Direct metabolic and redox observations support NE as a regulator of selected immune-cell responses. The proposed connection from these changes to immunoparalysis, secondary infection, and organ dysfunction defines a translational hypothesis whose cellular mediation and clinical effect require testing. Balancing the hemodynamic benefits of NE with its potential immunometabolic costs may help move vasopressor therapy in septic shock from a single MAP target toward more individualized management. **Figure 3** summarizes this framework, distinguishing experimentally observed cellular effects from proposed regulatory and clinical links. Future studies should pair standardized NE exposure with cell-specific metabolism, microbial killing, infection outcomes, and perfusion safety.

**Figure 3:**
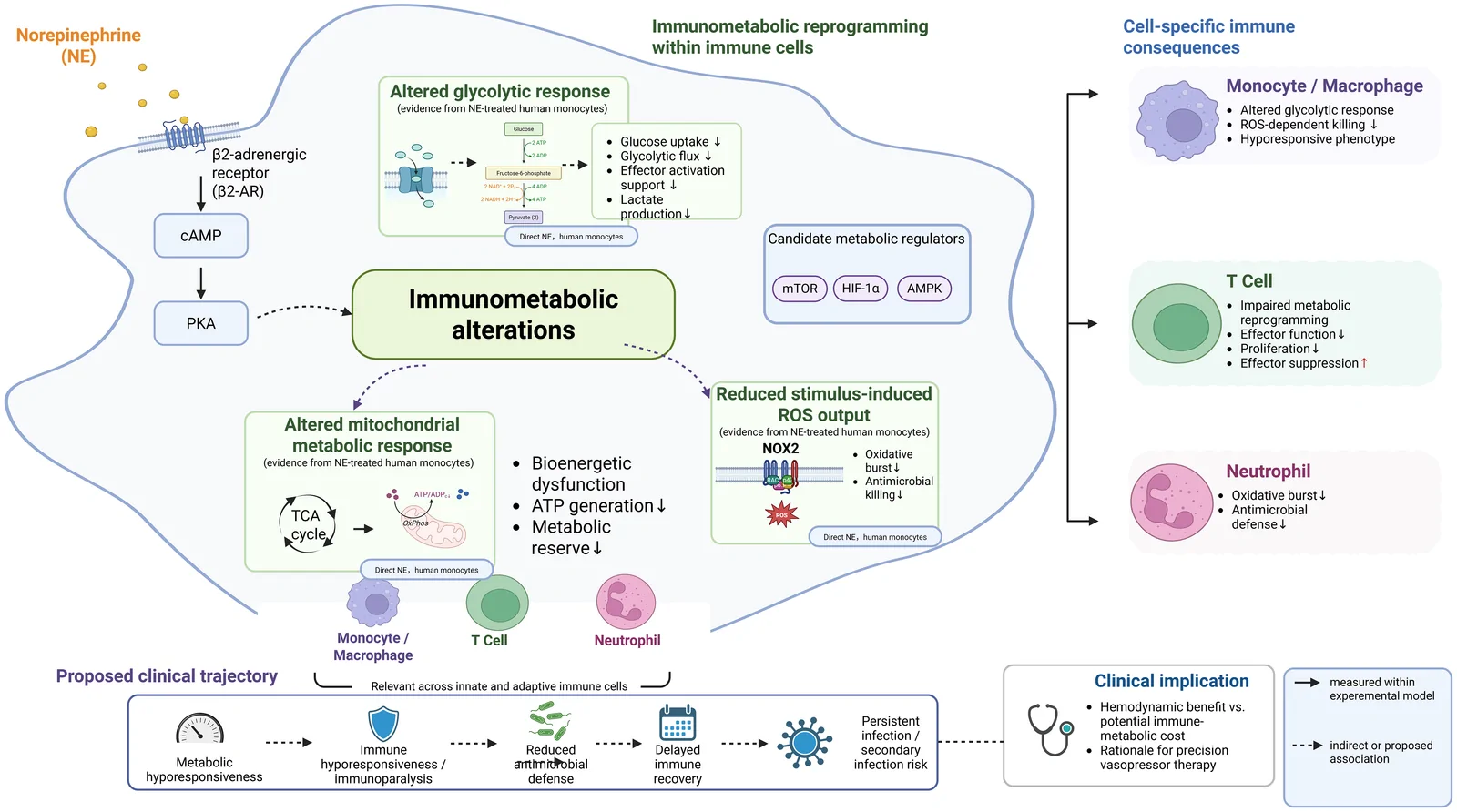
Norepinephrine-associated immunometabolic alterations and their potential immune and clinical consequences in septic shock. Norepinephrine (NE) engages β_2_-adrenergic receptor (β_2_-AR)/cAMP/protein kinase A (PKA) signaling, which may contribute to immune-cell metabolic changes. Direct NE experiments in human monocytes support attenuated LPS-induced lactate production, reduced spare respiratory capacity and maximal ECAR, and decreased stimulus-induced ROS output under defined conditions. Reduced glucose uptake, ATP generation, and antimicrobial killing are proposed functional interpretations requiring separate confirmation. NOX2 illustrates oxidative-burst machinery; its depiction does not establish direct inhibition by NE or a uniform reduction in mitochondrial ROS. T-cell metabolic findings include evidence from related β_2_-AR agonist models. Responses vary with cell type, activation state, and exposure conditions. HIF-1α, AMPK, and mTOR represent candidate metabolic regulators, without implying a linear cascade or established NE dependence in septic immune cells. The lower panel presents a proposed trajectory linking metabolic hyporesponsiveness to immune dysfunction, impaired antimicrobial defense, delayed immune recovery, and persistent or secondary infection. This clinical trajectory remains incompletely validated. Solid arrows indicate established signaling relationships or effects measured within the indicated experimental models; dashed arrows indicate indirect or proposed associations. Potential immune-metabolic costs are considered alongside the hemodynamic benefits of NE as a rationale for precision vasopressor therapy. cAMP, cyclic adenosine monophosphate; LPS, lipopolysaccharide; ECAR, extracellular acidification rate; ATP, adenosine triphosphate; ROS, reactive oxygen species; NOX2, NADPH oxidase 2; TCA, tricarboxylic acid; OXPHOS, oxidative phosphorylation; HIF-1α, hypoxia-inducible factor 1-alpha; AMPK, AMP-activated protein kinase; mTOR, mechanistic target of rapamycin. Created in BioRender. Yan, Xiao. (2026) https://BioRender.com/7fo7vh2.

## 5 Clinical implications of the immune effects of norepinephrine

The core clinical value of NE in septic shock remains the restoration of vascular tone, maintenance of MAP, and improvement of vital organ perfusion. In patients with persistent hypotension and tissue hypoperfusion, timely NE use can shorten shock duration, reduce excessive fluid resuscitation, and provide time for antimicrobial therapy and organ support (9). The CENSER trial showed that early NE improved early shock control, but it did not establish a definitive benefit for mortality or other patient-centered outcomes (11). Therefore, discussing the immune effects of NE is not intended to challenge its hemodynamic value. It aims to provide a more complete assessment of its clinical impact beyond circulatory support.

NE can affect cytokine release, phagocytic killing, antigen presentation, oxidative responses, and cellular metabolic states through adrenergic receptors. These effects may limit inflammatory tissue injury during acute inflammation. Experimental evidence suggests that during persistent infection, immunoparalysis, or prolonged high-dose exposure, they may also weaken host defense (14). The key clinical question has therefore shifted. It is no longer only whether NE has immune effects. It is which patients, at what dose, and during which exposure window may experience an immune cost alongside hemodynamic benefit.

### 5.1 Norepinephrine and the risk of secondary infection

Secondary infection is an important cause of poor outcomes after sepsis. It is also one of the most clinically relevant manifestations of immunoparalysis. Pathologic studies have shown that patients who die from sepsis and multiple organ failure often have persistent immunosuppression. This includes impaired antigen presentation, lymphocyte exhaustion, and activation of immune checkpoint pathways (71). Clinically, some patients survive early shock and inflammatory injury. However, they may later develop hospital-acquired infections, ventilator-associated pneumonia, catheter-related bloodstream infections, or opportunistic infections. These events reflect immune dysfunction but are also shaped by disease severity, pathogen burden, invasive devices, antimicrobial exposure, and source control.

mHLA-DR is an important marker for assessing immune hyporesponsiveness in sepsis. Leijte et al. showed that mHLA-DR kinetics were associated with infection site, pathogen type, and adverse outcomes (72). A recent real-world cohort including 1,023 patients with septic shock further showed that low mHLA-DR was associated with mortality and ICU-acquired infection (73). These findings support immune dysfunction as an important component of secondary-infection risk, although mHLA-DR is not specific to NE exposure and infection risk remains multifactorial.

NE may amplify this process. Translational studies have shown that NE can reduce pro-inflammatory mediator release, enhance IL-10 responses, and impair oxidative killing by monocytes and neutrophils. In polymicrobial sepsis models, sustained NE exposure was associated with increased bacterial dissemination (14). These findings support a biologically plausible NE-host defense interaction in experimental settings, but do not establish that therapeutic NE independently causes secondary infection in patients.

Clinical causality should be interpreted cautiously. Patients who receive high-dose NE are usually more severely ill. They often have more severe circulatory failure, higher infection burden, greater organ dysfunction, and more profound immune dysregulation. Associations between NE dose and secondary infection or mortality may reflect both drug exposure and disease severity. A more reasonable interpretation is that NE may act as an amplifier in patients with preexisting immunoparalysis, poor infection control, and high catecholamine burden. Future studies should jointly analyze NE exposure, immune phenotype, and infection outcomes. Grouping patients only by whether NE is used cannot answer the key clinical question.

### 5.2 Norepinephrine and pathogen behavior

The clinical effects of NE may extend to host-pathogen interactions. Studies in microbial endocrinology suggest that some pathogens can sense host stress-related neurotransmitters. They may use these signals to regulate growth, iron acquisition, adhesion, quorum sensing, and virulence expression (74). During severe infection and shock, catecholamine signaling is enhanced in local tissues, blood, and infectious microenvironments. These signals may influence pathogen adaptation, but their relevance at clinical NE concentrations remains uncertain.

One important mechanism by which NE may promote pathogen growth is related to iron acquisition. Many bacteria face iron restriction within the host. NE can promote iron acquisition in some bacteria from host iron-binding proteins such as transferrin and lactoferrin. This can support pathogen proliferation (75). Subsequent studies further showed that catecholamines can alter the iron-binding state of transferrin and lactoferrin. Under *in vitro* conditions, this can increase access to otherwise restricted iron (76); whether the same effect occurs during clinical NE infusion is unknown.

NE may also affect virulence-related behaviors and colonization in selected pathogens. In Pseudomonas aeruginosa, Enterobacteriaceae, and some Gram-positive bacteria, catecholamine stimulation has been associated with changes in quorum sensing, adhesion, biofilm formation, and virulence-related gene expression (74). These findings may help explain persistent infection, pathogen adaptation, and failure of antimicrobial therapy. However, current evidence mainly comes from animal experiments and *in vitro* studies. It cannot be directly inferred that clinical NE infusion necessarily enhances pathogen virulence in patients. Pathogen species, infection site, local NE concentration, antimicrobial exposure, and host immune status may all influence the final outcome.

### 5.3 Norepinephrine and bacterial biofilm formation

Biofilm formation is an important cause of persistent ICU infection and antimicrobial treatment failure. Mechanical ventilation, central venous catheters, urinary catheters, drainage tubes, and other invasive devices provide conditions for pathogen adhesion and biofilm formation. Bacteria within biofilms are enclosed by an extracellular polymeric matrix. This limits antimicrobial penetration and reduces immune-cell recognition and phagocytosis. As a result, pathogens are more likely to form persistent infectious foci.

Catecholamine signaling may participate in biofilm formation. Classic studies showed that catecholaminergic inotropes can promote Staphylococcus epidermidis growth, iron acquisition, and biofilm formation. This suggests that catecholamines may help shape the microenvironment of catheter-related and device-related infections (20). Recent studies have also found that epinephrine and NE can affect biofilm formation and adhesion in some Enterococcus strains. These effects are strain dependent and influenced by experimental conditions (77).

Direct evidence linking NE to clinical biofilm infection remains limited. It has not been proven that routine-dose NE infusion directly induces biofilm formation in patients. A more cautious interpretation is that catecholamine signaling may participate in shaping infectious microenvironments. Under specific pathogen, device-related infection, and host immune conditions, it may influence pathogen persistence. Further studies should integrate clinical samples, local drug concentrations, pathogen omics, and biofilm phenotypes.

### 5.4 Norepinephrine and organ dysfunction

The effects of NE on organ function are dual. During circulatory failure, NE can improve effective perfusion of the coronary circulation, brain, kidneys, and other vital organs by restoring perfusion pressure and maintaining vascular tone. Delayed vasopressor therapy prolongs hypotension and hypoperfusion. This can worsen tissue hypoxia, increase lactate levels, and aggravate multiple organ dysfunction. Therefore, the organ-protective effect of NE first derives from its hemodynamic value.

Achieving MAP targets does not mean that microcirculation and cellular oxygen utilization recover at the same time. Septic shock involves endothelial injury, glycocalyx degradation, microthrombus formation, abnormal leukocyte-endothelial interactions, and heterogeneous capillary perfusion. Even when macrocirculatory indices improve, local tissues may still have impaired oxygen delivery and utilization (78). Under some conditions, high-dose NE may increase vasoconstriction in certain microvascular beds and make tissue perfusion more heterogeneous. In patients with severe endothelial injury, microcirculatory dysfunction, or poor peripheral perfusion, simply pursuing a higher MAP may not improve tissue oxygenation.

Immunometabolic dysfunction may also affect organ recovery. Sepsis-associated mitochondrial dysfunction and redox imbalance may delay tissue repair; the independent contribution of therapeutic NE to this process remains uncertain (57). When hypotension remains uncorrected, NE is often necessary to prevent further organ hypoperfusion. The clinical challenge is to avoid two risks at the same time. Delaying necessary vasopressor therapy can aggravate hypoperfusion injury. Continuing unnecessary high-dose catecholamine exposure after tissue perfusion has stabilized may also create additional costs.

### 5.5 Norepinephrine exposure intensity and patient outcomes

Clinical studies often use norepinephrine equivalent dose, vasoactive drug scores, or vasoactive drug exposure indices to assess catecholamine burden. In recent years, attention has shifted from NE dose at a single time point to the overall burden defined by dose, duration, and cumulative exposure. Compared with a single peak dose, sustained high-dose exposure may better reflect a prolonged state of severe circulatory failure and high stress.

A systematic review and dose-response meta-analysis showed that higher NE doses were associated with increased mortality risk. However, the authors also emphasized that NE dose is an important marker of circulatory failure severity (79). These findings are useful for risk stratification but should not be interpreted as evidence that higher NE doses directly increase mortality (80). NE dose is usually strongly associated with disease severity. It reflects shock severity, reduced vascular responsiveness, infection burden, and inflammatory injury.

NE exposure intensity may be more useful as a risk stratification marker. Clinically, attention should be paid to NE dose trajectory, exposure duration, response to fluids and combined vasopressors, and the presence of poor lactate clearance, poor peripheral perfusion, immunoparalysis, or secondary infection. In recent years, the heterogeneity of NE dose conversion and reporting has also received attention. Studies have shown that different NE salts or molecular reference standards can affect mortality prediction. This highlights the need for standardized methods of NE dose reporting in future studies (80). If future research integrates NE exposure trajectories with immune monitoring, metabolomics, microcirculatory indices, and infection outcomes, it may better distinguish high NE requirements driven by disease severity from additional risks related to excessive catecholamine exposure.

### 5.6 Clinical controversies and the direction of precision vasopressor therapy

The clinical significance of NE-related immune effects remains debated. Supportive views emphasize that NE may promote anti-inflammatory responses, impair pathogen clearance, affect immunometabolism, and alter pathogen behavior. Under sustained exposure and susceptible immune phenotypes, these effects could amplify immunoparalysis and secondary-infection risk, but this remains unconfirmed clinically. Skeptical views emphasize that much of the current evidence comes from *in vitro* experiments or animal models. In clinical patients, NE exposure and immune outcomes are strongly confounded. Current evidence is not sufficient to conclude that NE directly worsens infection. This debate reflects the complexity of septic shock research. NE has clear life-saving value, but it may also produce non-hemodynamic effects under specific conditions.

Reducing unnecessary catecholamine exposure has gradually become an important concept in septic shock treatment. Relevant strategies include optimizing fluid resuscitation, setting appropriate MAP targets, identifying vascular hyporesponsiveness early, and using vasopressin or other adjunctive vasoactive agents at appropriate times (33, 81). The core aim of these strategies is to reduce potential additional risks from prolonged high-dose catecholamine exposure while ensuring tissue perfusion. Specific drug selection, timing of combination therapy, and target populations should still be determined according to circulatory status, cardiac function, vascular responsiveness, and shock stage.

In this context, vasopressor therapy is moving from a single MAP target toward individualized management that integrates tissue perfusion, catecholamine exposure intensity, and immune status. Treatment goals can be adjusted according to baseline blood pressure, lactate kinetics, urine output, skin perfusion, microcirculatory status, cardiac function, vascular responsiveness, and immune phenotype. In patients with marked immunoparalysis, high secondary infection risk, or prolonged high-dose NE requirements, early identification of high catecholamine burden may be especially important. These measures may characterize immune and perfusion status in research settings, but they are not NE-specific and cannot currently guide NE dosing.

The clinical significance of NE-related immune effects lies in shifting vasopressor therapy from a single MAP target toward maintaining effective tissue perfusion with the minimum necessary catecholamine exposure. Integrating circulatory support, immune status, and infection control may help explain why some patients with septic shock continue to develop immunosuppression, persistent infection, and poor organ recovery after macrocirculatory improvement. This approach may also provide a translational basis for precision vasopressor therapy and decatecholaminization strategies.

## 6 Potential strategies to reduce norepinephrine-related immunosuppression and precision vasopressor therapy

The primary goal of septic shock treatment remains correction of hypotension, restoration of tissue perfusion, and prevention of progressive organ injury. NE has a central role in this process. However, advances in neuroimmunology and immunometabolism have expanded the biological meaning of NE beyond vasoconstriction. NE may also participate in immune regulation, metabolic remodeling, and host-pathogen interactions. The concept of high catecholamine burden does not weaken the clinical value of NE. Instead, it indicates that vasopressor therapy should consider perfusion benefit, exposure intensity, and patient immune status at the same time (14).

Accordingly, septic shock management is moving from a single MAP target toward integrated resuscitation. Reasonable treatment goals should include rapid restoration of blood pressure. They should also consider the duration of high-dose NE exposure, vascular hyporesponsiveness, tissue perfusion quality, and immune phenotype. Precision vasopressor therapy aims to use NE in the right patient, at the right stage, and at the lowest effective dose. It also aims to balance circulatory support, immune homeostasis, and metabolic recovery.

### 6.1 Concept of decatecholaminization

For many years, circulatory support in septic shock has focused on blood pressure restoration and vasopressor response. NE rapidly increases MAP, improves coronary and renal perfusion pressure, and reduces tissue injury caused by persistent hypoperfusion. In early shock, delayed NE use may prolong hypotension and worsen organ dysfunction. Therefore, any strategy that reduces catecholamine exposure must first ensure adequate tissue perfusion.

The concept of catecholamine toxicity has led to renewed attention to sustained adrenergic stress during critical illness. High-dose or prolonged NE use often reflects disease severity. It may also be associated with increased myocardial oxygen consumption, arrhythmias, excessive peripheral vasoconstriction, heterogeneous microcirculatory perfusion, metabolic stress, and abnormal immune regulation (82). NE-related risk depends on dose trajectory, exposure duration, concomitant drugs, and baseline patient condition. A single dose at one time point cannot fully reflect the true exposure burden. A recent dose-response meta-analysis showed that higher NE doses were associated with increased mortality risk. However, this association is also influenced by disease severity, shock duration, and vascular responsiveness (79).

Decatecholaminization emphasizes reducing unnecessary exogenous catecholamine burden while maintaining effective perfusion. Its purpose is to avoid long-term dependence on a single high-dose NE strategy. This is especially relevant when tissue perfusion has partially improved, vascular hyporesponsiveness is evident, or alternative vasopressor pathways are available. A practical approach is to use the lowest NE dose that maintains perfusion, guided by shock stage, cardiac function, vascular tone, and fluid responsiveness; immune status remains an investigational consideration. When needed, non-catecholaminergic vasopressors or other adjunctive therapies can be combined. Recent studies also show that differences in NE dose reporting can affect risk assessment. This supports standardized reporting of NE exposure and equivalent doses in future studies (80).

### 6.2 Combining non-catecholaminergic vasopressors to reduce NE exposure

Early combination with non-catecholaminergic vasopressors is an important strategy to reduce NE dependence. Vasodilation in septic shock involves relative vasopressin deficiency, dysregulation of the renin-angiotensin system, excessive nitric oxide production, vascular smooth muscle hyporesponsiveness, and endothelial dysfunction. It is not driven only by adrenergic pathways. Targeting different vasoconstrictive pathways may reduce the burden of single-pathway adrenergic stimulation. A recent meta-analysis of randomized trials suggested that non-adrenergic vasopressors can reduce adrenergic exposure, whereas effects on clinical outcomes vary across agents, shock types, and initiation timing (32).

Vasopressin is the most extensively studied NE-sparing vasopressor. Patients with septic shock may develop relative vasopressin deficiency. Exogenous supplementation therefore has a clear pathophysiologic rationale. The VASST trial showed that low-dose vasopressin did not significantly reduce overall mortality compared with NE. Vasopressin remains an established adjunct to NE, but subgroup signals in VASST should be considered exploratory rather than evidence of a general survival benefit (83).

The VANISH trial further evaluated early vasopressin versus NE in septic shock. Vasopressin did not improve kidney failure-free days compared with NE. It therefore should not be interpreted as a proven renal-protective replacement for NE. However, it remains an NE-sparing strategy in early septic shock, and its renal effects require further evaluation (84).

The timing of vasopressin may be more important than its use alone. Observational studies suggest that catecholamine dose, lactate level, and shock duration at vasopressin initiation are associated with mortality risk. These observational associations support prospective evaluation of earlier vasopressin initiation but do not establish an optimal treatment threshold (85). A 2025 study based on large public databases also suggested that earlier vasopressin combination may be associated with lower in-hospital mortality. However, causal inference remains limited because of the observational design (86).

Angiotensin II provides another non-adrenergic pathway for refractory vasodilatory shock. The ATHOS-3 trial showed that angiotensin II increased the proportion of patients with refractory vasodilatory shock who achieved a MAP response. It also reduced the need for other vasopressors (87). In patients with acute kidney injury receiving renal replacement therapy, a *post hoc* analysis suggested that angiotensin II may be associated with renal recovery. This finding should be interpreted cautiously because of study design and patient selection (88). Recent reviews suggest that angiotensin II may be more suitable for patients with high NE requirements, poor vascular responsiveness, or selected renin-angiotensin system abnormalities (89).

Overall, non-catecholaminergic vasopressors are best viewed as part of multi-pathway vasopressor management. NE rapidly restores vascular tone and perfusion pressure. Vasopressin or angiotensin II may reduce adrenergic stimulation burden in selected patients. Future precision vasopressor strategies may move away from fixed stepwise escalation. They may instead use early or sequential combination therapy according to vascular hyporesponsiveness, catecholamine burden, perfusion status, and immune risk.

### 6.3 Individualized blood pressure targets and precision perfusion assessment

Current septic shock treatment usually uses a MAP of 65 mmHg as the initial target. This target is simple, practical, and suitable as an early safety threshold (9). In clinical practice, however, perfusion pressure requirements differ across patients. Patients with chronic hypertension, chronic kidney disease, atherosclerosis, cardiac dysfunction, or severe microcirculatory abnormalities may need different perfusion pressures. A uniform MAP target may be insufficient for some patients. In others, it may lead to unnecessary high-dose NE exposure.

The SEPSISPAM trial suggested that a higher MAP target did not improve overall survival. However, it may have reduced acute kidney injury in patients with chronic hypertension (90). The 65 Trial showed that a permissive lower MAP target reduced vasopressor exposure in older patients with vasodilatory hypotension, without a statistically significant difference in 90-day mortality (91). These findings support adjustment of MAP targets according to baseline blood pressure, age, renal risk, and vasopressor requirement. Simply pursuing a higher blood pressure does not necessarily provide greater benefit.

Tissue perfusion assessment is changing how vasopressor therapy is judged. After the MAP target is achieved, patients may still have poor lactate clearance, skin mottling, low urine output, prolonged capillary refill time (CRT), increased venous-to-arterial carbon dioxide difference, or microcirculatory dysfunction. In this setting, further increasing the NE dose may not improve tissue oxygenation. It may worsen local perfusion heterogeneity. In contrast, if MAP is slightly lower but CRT is normal, lactate decreases, urine output recovers, and peripheral perfusion improves, stronger vasopressor escalation may not be necessary.

CRT has received increasing attention because it is simple, rapid, and repeatable at the bedside. The ANDROMEDA-SHOCK trial showed that CRT-guided resuscitation did not significantly reduce 28-day mortality compared with lactate-guided resuscitation. However, it was associated with faster improvement in organ dysfunction (92). The ANDROMEDA-SHOCK-2 trial further combined CRT with an individualized hemodynamic algorithm. This strategy improved a hierarchical composite outcome that included mortality, duration of organ support, and length of hospital stay (93). These results provide more direct clinical support for individualized perfusion management.

The goal of precision perfusion management is to identify the lowest effective perfusion pressure for the current disease stage. During early severe hypoperfusion, MAP and organ perfusion should be restored promptly. After circulatory stabilization, treatment can gradually shift toward reducing catecholamine burden, assessing microcirculatory recovery, and avoiding excessive vasopressor escalation. Bedside ultrasound, pulse pressure variation, arterial elastance, microcirculatory imaging, and tissue oxygen monitoring may further refine this approach. Combining MAP, lactate, urine output, CRT, skin perfusion, and cardiac function may help avoid continued NE escalation driven by a single blood pressure value.

### 6.4 Precision vasopressor therapy guided by immune monitoring

Current vasopressor therapy is mainly adjusted according to hemodynamic variables. Patient immune status is rarely incorporated into dosing decisions. This is a major limitation. Immune responses in sepsis are highly heterogeneous. Some patients are dominated by hyperinflammation. Others develop early immunoparalysis, impaired antigen presentation, and lymphocyte exhaustion. The same NE dose may have different consequences across immune phenotypes. Experimental evidence suggests that the immune effects of sustained catecholamine exposure may differ between immune-hyporesponsive and hyperinflammatory states, but immune phenotype has not been validated for NE titration in clinical practice.

mHLA-DR is one of the immune monitoring markers closest to clinical application. Persistently low mHLA-DR usually indicates impaired monocyte antigen-presenting capacity. It is associated with secondary infection, ICU-acquired infection, and poor outcomes (73). PD-1/PD-L1, IL-10, lymphocyte counts, T-cell function, monocyte cytokine responses, and neutrophil killing capacity may also help identify an immunosuppressive state. Combining these markers with NE exposure trajectories may help test which immune phenotypes are most susceptible to sustained catecholamine exposure, but it cannot currently determine when NE should be reduced.

Transcriptomic and multi-omics technologies are advancing immune phenotyping in sepsis. Scicluna et al. identified sepsis endotypes based on blood gene expression. These endotypes differed in immune activity, organ dysfunction, and mortality risk (94). Seymour et al. derived and validated clinical sepsis phenotypes. These phenotypes were associated with differences in host response and potential treatment effects (95). Recent global multi-cohort transcriptomic studies further showed that host gene expression can identify sepsis endotypes across regions and pathogen backgrounds (28). If these endotypes can be translated into reproducible bedside markers, their value should be tested prospectively alongside blood pressure, perfusion, and treatment-response trajectories.

Immune monitoring-based precision vasopressor therapy can use a two-dimensional framework. One dimension assesses circulatory failure. This includes MAP, lactate, urine output, CRT, vascular responsiveness, and cardiac function. The other assesses immune risk. This includes mHLA-DR, lymphopenia, IL-10, PD-1/PD-L1, and leukocyte metabolic function. Patients with severe circulatory failure and preserved immune function may derive greater net benefit from intensified NE therapy. Patients with improving circulation but marked immunoparalysis and persistent high NE requirements may require earlier catecholamine reduction and combination with other vasopressor pathways.

This model remains conceptual and translational. No randomized trial has yet shown that adjusting NE dose according to immune markers improves outcomes. Its value lies in providing a testable pathway for future clinical studies. Future trials should evaluate cumulative NE exposure, immune phenotype changes, secondary infection, and organ recovery together. This may allow a more accurate assessment of the immune cost of vasopressor therapy.

Taken together, these strategies suggest that precision vasopressor therapy should not be limited to achieving a fixed MAP target or reducing NE dose alone. Instead, vasopressor management may need to integrate hemodynamic response, catecholamine exposure trajectory, tissue perfusion, vascular responsiveness, immune phenotype, and potential immunometabolic vulnerability. The major precision vasopressor and catecholamine-sparing strategies discussed in this review are summarized in **Table 2**.

**Table 2:** Precision vasopressor and catecholamine-sparing strategies in septic shock.

Strategy	Operational focus	Hemodynamic rationale	Precision relevance	Key caution	Ref
Early NE with fluid stewardship	Early vasoplegia	Restore MAP	Shorten hypotension burden	Mortality benefit uncertain	(10, 11, 31, 81)
	Fluid intolerance risk	Limit fluid overload	Avoid over-resuscitation	Avoid excessive cumulative NE	(10, 11, 31, 81)
Vasopressin add-on	NE-dependent vasodilatory shock	V1-mediated vascular tone	NE-sparing effect	Outcome benefit inconsistent	(32, 83–86)
	High adrenergic exposure	Non-β-adrenergic support	Lower β-AR stimulation	Timing and dose matter	(32, 83–86)
Angiotensin II	Refractory vasodilatory shock	AT1-mediated vascular tone	Catecholamine-sparing option	Careful patient selection	(87–89)
	Possible RRT-related phenotype	Rapid MAP response	Renin-guided interest	Monitor thrombotic risk	(87–89)
Individualized MAP target	Chronic hypertension/vascular risk	Avoid fixed MAP escalation	Reduce unnecessary NE exposure	Preserve organ perfusion	(90, 91)
	Perfusion-pressure tailoring	Balance MAP and flow	MAP–perfusion matching	High MAP may increase NE load	(90, 91)
Peripheral perfusion/CRT guidance	Assessable skin perfusion	Target tissue perfusion	Hemodynamic coherence	Requires training	(78, 92, 93)
	Lactate–perfusion mismatch	Avoid lactate-only escalation	CRT-guided adjustment	Not valid in all contexts	(78, 92, 93)
NE exposure metrics	Dose trajectory	Track vasopressor burden	NE-equivalent dose	Confounded by shock severity	(79–81)
	Exposure duration	Quantify cumulative load	Dose-time exposure	Affected by source control	(79–81)
β1-blockade after stabilization	Persistent tachycardia	Heart-rate control	Reduce adrenergic stress	Use only after stabilization	(82, 105, 106)
	Myocardial oxygen demand	Improve cardiac efficiency	HR–perfusion matching	Trial results mixed	(82, 105, 106)
α2-agonist sympatholysis	Sedated adrenergic phenotype	Central sympatholysis	Lower sympathetic tone	Hypotension/bradycardia	(107)
	Sedation strategy	Possible NEq/MAP reduction	Catecholamine-burden modulation	Evidence exploratory	(107)
Immune/endotype-guided strategy	Immunoparalysis phenotype	Avoid exposure-only decisions	mHLA-DR; IL-10	Not validated for NE titration	(72, 73, 94, 95, 97–101)
	Hyperinflammatory phenotype	Match support to immune state	PD-1/PD-L1; transcriptomics	Requires prospective testing	(72, 73, 94, 95, 97–101)
Catecholamine-sparing bundle	High NE dose/prolonged shock	Combine non-adrenergic support	Balance perfusion and immune cost	Conceptual framework	(32, 79, 87, 92, 97, 98, 101)
	Vasopressor resistance	Integrate MAP, CRT, and immune monitoring	Precision vasopressor strategy	Needs clinical validation	(32, 79, 87, 92, 97, 98, 101)

Beyond reducing unnecessary catecholamine exposure, another important question is whether established immunometabolic dysfunction can be directly corrected. The following section discusses potential strategies targeting immune-cell metabolism, mitochondrial function, and redox balance in septic shock.

### 6.5 Targeting immunometabolic abnormalities

NE-associated immune effects may involve immunometabolic remodeling, although direct clinical evidence linking therapeutic NE exposure to these changes remains limited. As discussed above, NE can affect monocyte glycolysis, oxidative phosphorylation, and ROS generation. It may also alter immune-cell activation thresholds through HIF-1α, AMPK, mTOR, and related metabolic nodes (17). Reducing NE exposure alone may not fully reverse established immunometabolic dysfunction. Restoring energy supply, mitochondrial function, and redox balance may better support host defense.

AMPK is an important node that connects energy stress, inflammation control, and mitochondrial homeostasis. Moderate AMPK activation can promote energy balance, autophagy regulation, and mitochondrial quality control. It may also reduce excessive inflammatory injury (96). AMPK-related interventions, including metformin, have been explored in early clinical research protocols for sepsis and sepsis-associated acute kidney injury. Current evidence is still insufficient to support routine use (70). Their potential value is better considered within a precision metabolic framework. Patient selection should consider metabolic status, renal function, lactate level, and immune phenotype.

Mitochondria-targeted therapy is another important direction. In sepsis, mitochondrial injury can lead to insufficient ATP generation, abnormal ROS regulation, mitochondrial DNA release, and persistent inflammatory signaling (57). Mitochondria-targeted antioxidants, interventions that promote mitochondrial biogenesis, mitophagy regulation, and NAD^+^ metabolic support may have potential value. At present, most evidence comes from animal experiments, cell studies, or early clinical exploration. Dose, timing, patient selection, and safety still need to be clarified.

NAD^+^ precursor supplementation provides another possible way to restore cellular energy metabolism. NAD^+^ participates in redox reactions, mitochondrial function, sirtuin activity, and DNA repair. During severe infection and persistent inflammation, increased NAD^+^ consumption may worsen energy deficiency in immune cells. Supplementing NAD^+^ precursors may theoretically improve cellular metabolic reserve and immune-cell responsiveness. However, its clinical efficacy in septic shock requires rigorous validation.

The key to immunometabolic intervention is to avoid a one-size-fits-all strategy. Metabolic needs differ across disease stages. During early hyperinflammation, suppressing some pro-inflammatory metabolic programs may reduce tissue injury. During immunoparalysis, restoring glycolytic adaptation, mitochondrial reserve, and oxidative killing capacity may be more important. Future clinical studies should stratify patients by immunometabolic phenotype. They should avoid placing all patients with sepsis into the same treatment pathway.

From a translational perspective, the most useful strategy may be to integrate three types of information: hemodynamic load, immune status, and metabolic adaptability. NE dose and exposure duration reflect catecholamine burden. mHLA-DR and lymphocyte function reflect immune status. Leukocyte metabolic assays and mitochondrial markers remain research tools, whereas lactate kinetics are not specific measures of immune-cell metabolism. Joint assessment of these domains should therefore be evaluated prospectively before guiding vasopressor or metabolic therapy.

NE remains a foundational drug for early resuscitation in septic shock. However, its use should become more precise. Limiting potential NE-associated immune effects may involve multi-pathway vasopressor support and individualized perfusion targets; immune-guided dosing and metabolic intervention remain investigational. These approaches aim to reduce unnecessary high catecholamine burden while maintaining tissue perfusion. With advances in immune phenotyping, metabolomics, and bedside perfusion monitoring, septic shock treatment may gradually move from MAP-centered vasopressor therapy toward coordinated neuro-immune-metabolic management.

## 7 Conclusion and outlook

NE remains the first-line vasopressor for restoring vascular tone and perfusion in septic shock. This review proposes that sustained high catecholamine burden may also link hemodynamic support with neuroendocrine stress, immune dysfunction, and metabolic reprogramming (14). This framework may help explain why macrocirculatory recovery does not always coincide with infection control, immune recovery, or organ repair.

Through adrenergic receptors, NE may alter cytokine production, phagocytic killing, antigen presentation, and lymphocyte function. These immune effects may involve cell-specific changes in glycolysis, mitochondrial respiration, and ROS generation, with HIF-1α, AMPK, and mTOR acting as context-dependent regulatory nodes rather than a single linear pathway (17). We therefore position NE as a potential neuro-immune-metabolic hub linking shock stress to immunoparalysis and persistent infection.

Evidence for NE-related immunosuppression and immunometabolic remodeling is derived mainly from experimental models, human endotoxemia, and observational cohorts. In septic shock, NE exposure is closely linked to disease severity, vascular hyporesponsiveness, and organ dysfunction; associations with mortality or infection therefore do not prove an independent drug effect. Prospective studies should standardize dose trajectories and cumulative exposure and relate them to immune phenotype, secondary infection, and organ recovery. Immune-guided assessment remains investigational because assays and treatment thresholds are not yet standardized for bedside use (97).

Precision vasopressor therapy should retain MAP as an initial safety target while integrating serial perfusion and cardiac assessments, including lactate kinetics, urine output, CRT, skin perfusion, and bedside ultrasound. ANDROMEDA-SHOCK-2 supports personalized hemodynamic resuscitation based on CRT, but it did not test immune-guided NE reduction (93). Immune markers such as mHLA-DR, lymphocyte count, IL-10, PD-1/PD-L1, and leukocyte function may refine research stratification (98). They are not yet validated for vasopressor titration because bedside availability, turnaround time, and treatment thresholds remain unresolved (97).

Future trials should assess more than short-term MAP achievement or vasopressor dose. Standardized NE exposure should be linked to perfusion safety, immune recovery, adjudicated secondary infection, organ-support duration, and longer-term outcomes. ImmunoSep supports the feasibility of immune phenotype-guided therapy, but it did not establish a mortality benefit or evaluate immune-guided vasopressor dosing (99, 100).NE-focused trials must therefore test whether lower catecholamine exposure or earlier non-catecholaminergic combination therapy benefits specific phenotypes and disease stages. Sepsis subphenotyping and companion diagnostics may eventually support phenotype-guided vasopressor management, but require prospective validation (101).

NE remains essential for early septic shock resuscitation, but unnecessary exposure should be minimized once perfusion stabilizes. The next step is to prospectively test whether hemodynamic, immune, and metabolic phenotyping can identify patients who benefit from catecholamine-sparing strategies without compromising perfusion. Such evidence is required before immune or metabolic measurements can guide routine vasopressor decisions.

## References

[1] Singer M Deutschman CS Seymour CW Shankar-Hari M Annane D Bauer M . The third international consensus definitions for sepsis and septic shock (Sepsis-3). JAMA. (2016) 315:801–10. doi: 10.1001/jama.2016.0287 26903338 PMC4968574

[2] Rudd KE Johnson SC Agesa KM Shackelford KA Tsoi D Kievlan DR . Global, regional, and national sepsis incidence and mortality, 1990-2017: Analysis for the Global Burden of Disease Study. Lancet. (2020) 395:200–11. doi: 10.1016/S0140-6736(19)32989-7 31954465 PMC6970225

[3] Cecconi M Evans L Levy M Rhodes A . Sepsis and septic shock. Lancet. (2018) 392:75–87. doi: 10.1016/S0140-6736(18)30696-2 29937192

[4] van der Poll T Shankar-Hari M Wiersinga WJ . The immunology of sepsis. Immunity. (2021) 54:2450–64. doi: 10.1016/j.immuni.2021.10.012 34758337

[5] Marshall JC . Why have clinical trials in sepsis failed? Trends Mol Med. (2014) 20:195–203. doi: 10.1016/j.molmed.2014.01.007 24581450

[6] Hotchkiss RS Monneret G Payen D . Sepsis-induced immunosuppression: From cellular dysfunctions to immunotherapy. Nat Rev Immunol. (2013) 13:862–74. doi: 10.1038/nri3552 24232462 PMC4077177

[7] Girardis M David S Ferrer R Helms J Juffermans NP Martin-Loeches I . Understanding, assessing and treating immune, endothelial and haemostasis dysfunctions in bacterial sepsis. Intensive Care Med. (2024) 50:1580–92. doi: 10.1007/s00134-024-07586-2 39222142

[8] Preau S Vodovar D Jung B Lancel S Zafrani L Flatres A . Energetic dysfunction in sepsis: A narrative review. Ann Intensive Care. (2021) 11:104. doi: 10.1186/s13613-021-00893-7 34216304 PMC8254847

[9] Prescott HC Antonelli M Alhazzani W Moller MH Alshamsi F Azevedo LCP . Surviving Sepsis Campaign: International guidelines for management of sepsis and septic shock 2026. Crit Care Med. (2026) 54:725–812. doi: 10.1097/CCM.0000000000007075 41869847

[10] Russell JA . Vasopressor therapy in critically ill patients with shock. Intensive Care Med. (2019) 45:1503–17. doi: 10.1007/s00134-019-05801-z 31646370

[11] Permpikul C Tongyoo S Viarasilpa T Trainarongsakul T Chakorn T Udompanturak S . Early use of norepinephrine in septic shock resuscitation (Censer). A randomized trial. Am J Respir Crit Care Med. (2019) 199:1097–105. doi: 10.1164/rccm.201806-1034OC 30704260

[12] Scanzano A Cosentino M . Adrenergic regulation of innate immunity: A review. Front Pharmacol. (2015) 6:171. doi: 10.3389/fphar.2015.00171 26321956 PMC4534859

[13] Stolk RF van der Poll T Angus DC van der Hoeven JG Pickkers P Kox M . Potentially inadvertent immunomodulation: Norepinephrine use in sepsis. Am J Respir Crit Care Med. (2016) 194:550–8. doi: 10.1164/rccm.201604-0862CP 27398737

[14] Stolk RF van der Pasch E Naumann F Schouwstra J Bressers S van Herwaarden AE . Norepinephrine dysregulates the immune response and compromises host defense during sepsis. Am J Respir Crit Care Med. (2020) 202:830–42. doi: 10.1164/rccm.202002-0339OC 32520577

[15] O'Neill LA Kishton RJ Rathmell J . A guide to immunometabolism for immunologists. Nat Rev Immunol. (2016) 16:553–65. doi: 10.1038/nri.2016.70 27396447 PMC5001910

[16] McBride MA Owen AM Stothers CL Hernandez A Luan L Burelbach KR . The metabolic basis of immune dysfunction following sepsis and trauma. Front Immunol. (2020) 11:1043. doi: 10.3389/fimmu.2020.01043 32547553 PMC7273750

[17] Thoppil J Mehta P Bartels B Sharma D Farrar JD . Impact of norepinephrine on immunity and oxidative metabolism in sepsis. Front Immunol. (2023) 14:1271098. doi: 10.3389/fimmu.2023.1271098 38022663 PMC10662053

[18] Flierl MA Rittirsch D Huber-Lang M Sarma JV Ward PA . Catecholamines-crafty weapons in the inflammatory arsenal of immune/inflammatory cells or opening Pandora's box? Mol Med. (2008) 14:195–204. doi: 10.2119/2007-00105.Flierl 18079995 PMC2136428

[19] Belfiore J Taddei R Biancofiore G . Catecholamines in sepsis: Pharmacological insights and clinical applications-a narrative review. J Anesth Analg Crit Care. (2025) 5:17. doi: 10.1186/s44158-025-00241-2 40176108 PMC11966821

[20] Lyte M Freestone PP Neal CP Olson BA Haigh RD Bayston R . Stimulation of Staphylococcus epidermidis growth and biofilm formation by catecholamine inotropes. Lancet. (2003) 361:130–5. doi: 10.1016/S0140-6736(03)12231-3 12531580

[21] Stolk RF Kox M Pickkers P . Noradrenaline drives immunosuppression in sepsis: Clinical consequences. Intensive Care Med. (2020) 46:1246–8. doi: 10.1007/s00134-020-06025-2 32270211 PMC7292809

[22] Akira S Uematsu S Takeuchi O . Pathogen recognition and innate immunity. Cell. (2006) 124:783–801. doi: 10.1016/j.cell.2006.02.015 16497588

[23] Gao X Cai S Li X Wu G . Sepsis-induced immunosuppression: Mechanisms, biomarkers and immunotherapy. Front Immunol. (2025) 16:1577105. doi: 10.3389/fimmu.2025.1577105 40364841 PMC12069044

[24] Monneret G Gossez M Aghaeepour N Gaudilliere B Venet F . How clinical flow cytometry rebooted sepsis immunology. Cytometry A. (2019) 95:431–41. doi: 10.1002/cyto.a.23749 30887636 PMC6461502

[25] Joshi I Carney WP Rock EP . Utility of monocyte Hla-Dr and rationale for therapeutic Gm-Csf in sepsis immunoparalysis. Front Immunol. (2023) 14:1130214. doi: 10.3389/fimmu.2023.1130214 36825018 PMC9942705

[26] Drewry AM Samra N Skrupky LP Fuller BM Compton SM Hotchkiss RS . Persistent lymphopenia after diagnosis of sepsis predicts mortality. Shock. (2014) 42:383–91. doi: 10.1097/SHK.0000000000000234 25051284 PMC4362626

[27] Zhang T Yu-Jing L Ma T . Role of regulation of Pd-1 and Pd-L1 expression in sepsis. Front Immunol. (2023) 14:1029438. doi: 10.3389/fimmu.2023.1029438 36969168 PMC10035551

[28] Chenoweth JG Brandsma J Striegel DA Genzor P Chiyka E Blair PW . Sepsis endotypes identified by host gene expression across global cohorts. Commun Med (Lond). (2024) 4:120. doi: 10.1038/s43856-024-00542-7 38890515 PMC11189468

[29] Carrara M Ferrario M Bollen Pinto B Herpain A . The autonomic nervous system in septic shock and its role as a future therapeutic target: A narrative review. Ann Intensive Care. (2021) 11:80. doi: 10.1186/s13613-021-00869-7 33999297 PMC8128952

[30] Van den Berghe G Teblick A Langouche L Gunst J . The hypothalamus-pituitary-adrenal axis in sepsis- and hyperinflammation-induced critical illness: Gaps in current knowledge and future translational research directions. EBioMedicine. (2022) 84:104284. doi: 10.1016/j.ebiom.2022.104284 36162206 PMC9519475

[31] Wieruszewski PM Khanna AK . Vasopressor choice and timing in vasodilatory shock. Crit Care. (2022) 26:76. doi: 10.1186/s13054-022-03911-7 35337346 PMC8957156

[32] Kotani Y Belletti A D'Amico F Bonaccorso A Wieruszewski PM Fujii T . Non-adrenergic vasopressors for vasodilatory shock or perioperative vasoplegia: A meta-analysis of randomized controlled trials. Crit Care. (2024) 28:439. doi: 10.1186/s13054-024-05212-7 39736782 PMC11686893

[33] Lajoye Q Orieux A Boyer A Prevel R Jozwiak M . Vasopressin and its analogues in patients with septic shock: Holy grail or unfulfilled promise? Crit Care. (2025) 29:333. doi: 10.1186/s13054-025-05540-2 40731360 PMC12306101

[34] Cheng S Scicluna B Arts R . Broad defects in the energy metabolism of leukocytes underlie immunoparalysis in sepsis. Nat Immunol. (2016) 17:406–13. doi: 10.1038/ni.3398 26950237

[35] Pavlov V Tracey K . Neural regulation of immunity: Molecular mechanisms and clinical translation. Nat Neurosci. (2017) 20:156–66. doi: 10.1038/nn.4477 28092663

[36] Chhatar S Lal G . Role of adrenergic receptor signalling in neuroimmune communication. Curr Res Immunol. (2021) 2:202–17. doi: 10.1016/j.crimmu.2021.11.001 35492402 PMC9040148

[37] Nguyen H Bastow C Wen S Wong C . Sympathetic regulation of the host immune response to bacterial sepsis. Clin Sci. (2025) 139:1215–37. doi: 10.1042/cs20256909 41124072 PMC12687452

[38] Sanders VM . The Beta2-adrenergic receptor on T and B lymphocytes: Do we understand it yet? Brain Behav Immun. (2012) 26:195–200. doi: 10.1016/j.bbi.2011.08.001 21855626 PMC3243812

[39] Thapa S Cao X . Nervous regulation: Beta-2-adrenergic signaling in immune homeostasis, cancer immunotherapy, and autoimmune diseases. Cancer Immunol Immunother. (2023) 72:2549–56. doi: 10.1007/s00262-023-03445-z 37060364 PMC10693916

[40] Farmer P Pugin J . B-adrenergic agonists exert their “anti-inflammatory” effects in monocytic cells through the Iκb/Nf-Kb pathway. Am J Physiol Lung Cell Mol Physiol. (2000) 279:L675–82. doi: 10.1152/ajplung.2000.279.4.L675 11000127

[41] Agac D Estrada LD Maples R Hooper LV Farrar JD . The Beta2-adrenergic receptor controls inflammation by driving rapid Il-10 secretion. Brain Behav Immun. (2018) 74:176–85. doi: 10.1016/j.bbi.2018.09.004 30195028 PMC6289674

[42] Lamkin DM Ho HY Ong TH Kawanishi CK Stoffers VL Ahlawat N . Beta-adrenergic-stimulated macrophages: Comprehensive localization in the M1-M2 spectrum. Brain Behav Immun. (2016) 57:338–46. doi: 10.1016/j.bbi.2016.07.162 27485040 PMC5011037

[43] Vida C Portilla Y Murga C . Adrenergic modulation of neutrophil and macrophage functions: Pathophysiological cues. Curr Opin Physiol. (2024) 41:100780. doi: 10.1016/j.cophys.2024.10078042574925

[44] Burn G Foti A Marsman G Patel D Zychlinsky A . The neutrophil. Immunity. (2021) 54:1377–91. doi: 10.1016/j.immuni.2021.06.006 34260886

[45] Maestroni G . Dendritic cell migration controlled by alpha 1b-adrenergic receptors. J Immunol. (2000) 165:6743–7. doi: 10.4049/jimmunol.165.12.6743 11120793

[46] Nijhuis L Olivier B Dhawan S . Adrenergic B2 receptor activation stimulates anti-inflammatory properties of dendritic cells *in vitro*. PloS One. (2014) 9:e85086. doi: 10.1371/journal.pone.0085086 24465481 PMC3898911

[47] Estrada L Ağaç D Farrar J . Sympathetic neural signaling via the B2-adrenergic receptor suppresses T-cell receptor-mediated human and mouse Cd8+ T-cell effector function. Eur J Immunol. (2016) 46:1948–58. doi: 10.1002/eji.201646395 27222010 PMC5241047

[48] Guereschi M Araujo L Maricato J . Beta2-adrenergic receptor signaling in Cd4+ Foxp3+ regulatory T cells enhances their suppressive function in a Pka-dependent manner. Eur J Immunol. (2013) 43:1001–12. doi: 10.1002/eji.201243005 23436577

[49] van der Heijden C Groh L Keating S . Catecholamines induce trained immunity in monocytes *in vitro* and *in vivo*. Circ Res. (2020) 127:269–83. doi: 10.1161/circresaha.119.315800 32241223

[50] Buck M Sowell R Kaech S Pearce E . Metabolic instruction of immunity. Cell. (2017) 169:570–86. doi: 10.1016/j.cell.2017.04.004 28475890 PMC5648021

[51] Pearce E Pearce E . Metabolic pathways in immune cell activation and quiescence. Immunity. (2013) 38:633–43. doi: 10.1016/j.immuni.2013.04.005 23601682 PMC3654249

[52] Van Wyngene L Vandewalle J Libert C . Reprogramming of basic metabolic pathways in microbial sepsis: Therapeutic targets at last? EMBO Mol Med. (2018) 10:e8712. doi: 10.15252/emmm.201708712 29976786 PMC6079534

[53] Palsson-McDermott E Curtis A Goel G . Pyruvate kinase M2 regulates Hif-1α activity and Il-1β induction and is a critical determinant of the Warburg effect in Lps-activated macrophages. Cell Metab. (2015) 21:65–80. doi: 10.1016/j.cmet.2014.12.005 25565206 PMC5198835

[54] Wasyluk W Zwolak A . Metabolic alterations in sepsis. J Clin Med. (2021) 10:2412. doi: 10.3390/jcm10112412 34072402 PMC8197843

[55] Qiao G Bucsek M Winder N Chen M Giridharan T Olejniczak S . B-adrenergic signaling blocks murine Cd8+ T-cell metabolic reprogramming during activation: A mechanism for immunosuppression by adrenergic stress. Cancer Immunol Immunother. (2019) 68:11–22. doi: 10.1007/s00262-018-2243-8 30229289 PMC6326964

[56] Ip W Hoshi N Shouval D Snapper S Medzhitov R . Anti-inflammatory effect of Il-10 mediated by metabolic reprogramming of macrophages. Science. (2017) 356:513–9. doi: 10.1126/science.aal3535 28473584 PMC6260791

[57] Hu D Sheeja Prabhakaran H Zhang Y . Mitochondrial dysfunction in sepsis: Mechanisms and therapeutic perspectives. Crit Care. (2024) 28:292. doi: 10.1186/s13054-024-05069-w 39227925 PMC11373266

[58] Shrestha D Pant B Roychowdhury S . Immunometabolic chaos in septic shock. J Leuk Biol. (2025) 117:qiae211. doi: 10.1093/jleuko/qiae211 39340428 PMC11879763

[59] Ma L Han T Zhan Y . Mechanism and role of mitophagy in the development of severe infection. Cell Death Discov. (2024) 10:88. doi: 10.1038/s41420-024-01844-4 38374038 PMC10876966

[60] Nathan C Cunningham-Bussel A . Beyond oxidative stress: An immunologist's guide to reactive oxygen species. Nat Rev Immunol. (2013) 13:349–61. doi: 10.1038/nri3423 23618831 PMC4250048

[61] Thoppil J Farrar J Sharma D Kirby S Mobley A Courtney D . Reactive oxygen species elevations in human immune cell subsets during sepsis are mitigated by norepinephrine and N-acetylcysteine. World J Crit Care Med. (2025) 14:108638. doi: 10.5492/wjccm.v14.i4.108638 41377542 PMC12687033

[62] McGettrick A O'Neill L . The role of Hif in immunity and inflammation. Cell Metab. (2020) 32:524–36. doi: 10.1016/j.cmet.2020.08.002 32853548

[63] O'Neill L Hardie D . Metabolism of inflammation limited by Ampk and pseudo-starvation. Nature. (2013) 493:346–55. doi: 10.1038/nature11862 23325217

[64] Huang J Liu K Zhu S Xie M Kang R Cao L . Ampk regulates immunometabolism in sepsis. Brain Behav Immun. (2018) 72:89–100. doi: 10.1016/j.bbi.2017.11.003 29109024

[65] Pollizzi K Powell J . Regulation of T cells by Mtor: The known knowns and the known unknowns. Trends Immunol. (2015) 36:13–20. doi: 10.1016/j.it.2014.11.005 25522665 PMC4290883

[66] Panwar V Singh A Bhatt M . Multifaceted role of Mtor signaling pathway in human health and disease. Signal Transduct Target Ther. (2023) 8:375. doi: 10.1038/s41392-023-01608-z 37779156 PMC10543444

[67] Langley R Tsalik E van Velkinburgh J . An integrated clinico-metabolomic model improves prediction of death in sepsis. Sci Transl Med. (2013) 5:195ra95. doi: 10.1126/scitranslmed.3005893 23884467 PMC3924586

[68] Chung K Chen G Chuang T . Increased plasma acetylcarnitine in sepsis is associated with multiple organ dysfunction and mortality: A multicenter cohort study. Crit Care Med. (2019) 47:210–8. doi: 10.1097/ccm.0000000000003517 30379669

[69] Chen Q Liang X Wu T . Integrative analysis of metabolomics and proteomics reveals amino acid metabolism disorder in sepsis. J Transl Med. (2022) 20:123. doi: 10.1186/s12967-022-03320-y 35287674 PMC8919526

[70] Saraiva I Hamahata N Huang D . Metformin for sepsis-associated Aki: Protocol for the randomized clinical trial of the safety and feasibility of metformin as a treatment for sepsis-associated Aki(Limit Aki). BMJ Open. (2024) 14:e081120. doi: 10.1136/bmjopen-2023-081120 38688665 PMC11086423

[71] Boomer J To K Chang K . Immunosuppression in patients who die of sepsis and multiple organ failure. JAMA. (2011) 306:2594–605. doi: 10.1001/jama.2011.1829 22187279 PMC3361243

[72] Leijte G Rimmelé T Kox M . Monocytic Hla-Dr expression kinetics in septic shock patients with different pathogens, sites of infection and adverse outcomes. Crit Care. (2020) 24:110. doi: 10.1186/s13054-020-2830-x 32192532 PMC7082984

[73] Monneret G Lafon T Gossez M . Monocyte Hla-Dr expression in septic shock patients: Insights from a 20-year real-world cohort of 1023 cases. Intensive Care Med. (2025) 51:1820–32. doi: 10.1007/s00134-025-08110-w 40986015 PMC12504386

[74] Boukerb A Cambronel M Rodrigues S . Inter-kingdom signaling of stress hormones: Sensing, transport and modulation of bacterial physiology. Front Microbiol. (2021) 12:690942. doi: 10.3389/fmicb.2021.690942 34690943 PMC8526972

[75] Freestone P Haigh R Williams P Lyte M . Involvement of enterobactin in norepinephrine-mediated iron supply from transferrin to enterohaemorrhagic Escherichia coli. FEMS Microbiol Lett. (2003) 222:39–43. doi: 10.1016/s0378-1097(03)00243-x 12757944

[76] Sandrini S Shergill R Woodward J . Elucidation of the mechanism by which catecholamine stress hormones liberate iron from the innate immune defense proteins transferrin and lactoferrin. J Bacteriol. (2010) 192:587–94. doi: 10.1128/jb.01028-09 19820086 PMC2805316

[77] Cambronel M Tortuel D Biaggini K . Influence of catecholamines(epinephrine/norepinephrine)on biofilm formation and adhesion in pathogenic and probiotic strains of Enterococcus faecalis. Front Microbiol. (2020) 11:1501. doi: 10.3389/fmicb.2020.01501 32849320 PMC7396564

[78] Ince C . Hemodynamic coherence and the rationale for monitoring the microcirculation. Crit Care. (2015) 19:S8. doi: 10.1186/cc14726 26729241 PMC4699073

[79] Reinikainen M Delamarre L Blaser A . Association of noradrenaline dose with mortality in critically ill patients: A systematic review and dose-response meta-analysis. Crit Care. (2025) 29:498. doi: 10.1186/s13054-025-05717-9 41267052 PMC12632045

[80] Morales S Wendel-Garcia P Ibarra-Estrada M . The impact of norepinephrine dose reporting heterogeneity on mortality prediction in septic shock patients. Crit Care. (2024) 28:216. doi: 10.1186/s13054-024-05011-0 38961499 PMC11220947

[81] Ammar M Ammar A Wieruszewski P . Timing of vasoactive agents and corticosteroid initiation in septic shock. Ann Intensive Care. (2022) 12:47. doi: 10.1186/s13613-022-01021-9 35644899 PMC9148864

[82] Dünser M Hasibeder W . Sympathetic overstimulation during critical illness: Adverse effects of adrenergic stress. J Intensive Care Med. (2009) 24:293–316. doi: 10.1177/0885066609340519 19703817

[83] Russell J Walley K Singer J . Vasopressin versus norepinephrine infusion in patients with septic shock. N Engl J Med. (2008) 358:877–87. doi: 10.1056/NEJMoa067373 18305265

[84] Gordon A Mason A Thirunavukkarasu N . Effect of early vasopressin vs norepinephrine on kidney failure in patients with septic shock: The Vanish randomized clinical trial. JAMA. (2016) 316:509–18. doi: 10.1001/jama.2016.10485 27483065

[85] Sacha G Lam S Wang L Duggal A Reddy A Bauer S . Association of catecholamine dose, lactate, and shock duration at vasopressin initiation with mortality in patients with septic shock. Crit Care Med. (2022) 50:614–23. doi: 10.1097/ccm.0000000000005317 34582425

[86] Sacha G Duggal A Reddy A Wang L Bauer S . Vasopressin initiation timing and in-hospital mortality in septic shock: An observational study of large public databases. Crit Care Explor. (2025) 7:e1284. doi: 10.1097/cce.0000000000001284 40844800 PMC12377302

[87] Khanna A English S Wang X . Angiotensin Ii for the treatment of vasodilatory shock. N Engl J Med. (2017) 377:419–30. doi: 10.1056/NEJMoa1704154 28528561

[88] Tumlin J Murugan R Deane A . Outcomes in patients with vasodilatory shock and renal replacement therapy treated with intravenous angiotensin Ii. Crit Care Med. (2018) 46:949–57. doi: 10.1097/ccm.0000000000003092 29509568 PMC5959265

[89] Coloretti I Genovese A Teixeira J . Angiotensin Ii therapy in refractory septic shock: Which patient can benefit most? A narrative review. J Anesthesia Analgesia Crit Care. (2024) 4:13. doi: 10.1186/s44158-024-00150-w 38383521 PMC10882873

[90] Asfar P Meziani F Hamel J . High versus low blood-pressure target in patients with septic shock. N Engl J Med. (2014) 370:1583–93. doi: 10.1056/NEJMoa1312173 24635770

[91] Lamontagne F Richards-Belle A Thomas K . Effect of reduced exposure to vasopressors on 90-day mortality in older critically ill patients with vasodilatory hypotension: A randomized clinical trial. JAMA. (2020) 323:938–49. doi: 10.1001/jama.2020.0930 32049269 PMC7064880

[92] Hernández G Ospina-Tascón G Damiani L . Effect of a resuscitation strategy targeting peripheral perfusion status vs serum lactate levels on 28-day mortality among patients with septic shock: The Andromeda-Shock randomized clinical trial. JAMA. (2019) 321:654–64. doi: 10.1001/jama.2019.0071 30772908 PMC6439620

[93] Hernández G Ospina-Tascón G Kattan E ANDROMEDA-SHOCK-2 Investigators . Personalized hemodynamic resuscitation targeting capillary refill time in early septic shock: The Andromeda-Shock-2 randomized clinical trial. JAMA. (2025) 334:1988–99. doi: 10.1001/jama.2025.20402 41159835 PMC12573117

[94] Scicluna B van Vught L Zwinderman A . Classification of patients with sepsis according to blood genomic endotype: A prospective cohort study. Lancet Respir Med. (2017) 5:816–26. doi: 10.1016/s2213-2600(17)30294-1 28864056

[95] Seymour C Kennedy J Wang S . Derivation, validation, and potential treatment implications of novel clinical phenotypes for sepsis. JAMA. (2019) 321:2003–17. doi: 10.1001/jama.2019.5791 31104070 PMC6537818

[96] Yumoto T Coopersmith C . Targeting Amp-activated protein kinase in sepsis. Front Endocrinol. (2024) 15:1452993. doi: 10.3389/fendo.2024.1452993 39469575 PMC11513325

[97] Cajander S Kox M Scicluna B . Profiling the dysregulated immune response in sepsis: Overcoming challenges to achieve the goal of precision medicine. Lancet Respir Med. (2024) 12:305–22. doi: 10.1016/s2213-2600(23)00330-2 38142698

[98] Rao M McGonagill P Brackenridge S . Functional immunophenotyping for precision therapies in sepsis. Shock. (2025) 63:189–201. doi: 10.1097/shk.0000000000002511 39617419 PMC12447363

[99] Giamarellos-Bourboulis E Kotsaki A Kotsamidi I ImmunoSep Study Group . Precision immunotherapy to improve sepsis outcomes: The Immunosep randomized clinical trial. JAMA. (2026) 335:775–86. doi: 10.1001/jama.2025.24175 41359996 PMC12687202

[100] Angus D . Precision therapy for sepsis: The end of the beginning? JAMA. (2026) 335:761–2. doi: 10.1001/jama.2025.24099 41359997

[101] Antcliffe D Burrell A Boyle A Gordon A McAuley D Silversides J . Sepsis subphenotypes, theragnostics and personalized sepsis care. Intensive Care Med. (2025) 51:756–68. doi: 10.1007/s00134-025-07873-6 40163135 PMC12055953

[102] Bellinger DL Lorton D . Autonomic regulation of cellular immune function. Autonomic Neuroscience: Basic Clin. (2014) 182:15–41. doi: 10.1016/j.autneu.2014.01.006 24685093

[103] Nance DM Sanders VM . Autonomic innervation and regulation of the immune system (1987-2007). Brain Behav Immun. (2007) 21:736–45. doi: 10.1016/j.bbi.2007.03.008 17467231 PMC1986730

[104] Nakai A Hayano Y Furuta F Noda M Suzuki K . Control of lymphocyte egress from lymph nodes through B2-adrenergic receptors. J Exp Med. (2014) 211:2583–98. doi: 10.1084/jem.20141132 25422496 PMC4267238

[105] Morelli A Ertmer C Westphal M Rehberg S Kampmeier T Ligges S . Effect of heart rate control with esmolol on hemodynamic and clinical outcomes in patients with septic shock: A randomized clinical trial. JAMA. (2013) 310:1683–91. doi: 10.1001/jama.2013.278477 24108526

[106] Whitehouse T Hossain A Perkins GD Gordon AC Bion J Young D . Landiolol and organ failure in patients with septic shock: The Stress-L randomized clinical trial. JAMA. (2023) 330:1641–52. doi: 10.1001/jama.2023.20134 37877587 PMC10600724

[107] Cioccari L Luethi N Bailey M Shehabi Y Howe B Messmer AS . The effect of dexmedetomidine on vasopressor requirements in patients with septic shock: A subgroup analysis of the sedation practice in intensive care evaluation [Spice Iii] trial. Crit Care. (2020) 24:441. doi: 10.1186/s13054-020-03115-x 32678054 PMC7367420

