# Supplementary material for: Norepinephrine in septic shock: catecholamine burden, immunometabolic reprogramming, and precision vasopressor therapy
Source: Front Immunol. 2026 Oct 22;17:1935801. doi: 10.3389/fimmu.2026.1935801 (PMC13736031; doi:10.3389/fimmu.2026.1935801)
Supplement: Supplementary file 1 [file DataSheet1.docx]

Supplementary Material

# 1 Supplementary Figures and Tables

**Supplementary Table S1 Key studies on norepinephrine and immunometabolic regulation**

| **Study / ref.** | **Study model** | **Evidence type** | **Agent and dose** | **Exposure / assessment** | **Measured endpoints** | **Main findings** | **Limitations and interpretation** |
| --- | --- | --- | --- | --- | --- | --- | --- |
| Stolk et al. 2020 (1) | Healthy-donor whole blood and primary monocytes. | Direct NE; in vitro. | NE 1 µM for metabolic assays; 0.01–10 µM for whole-blood cytokine dose series. | 1 h before LPS; cytokines/lactate at 24 h. Flux: 1 h exposure plus 1 h equilibration, with NE maintained. | Cytokines; lactate; ECAR; OCR and spare respiratory capacity. | LPS-induced TNF-α and lactate decreased; IL-10 increased. ECAR and spare respiratory capacity decreased in separate assays. | Direct immunometabolic evidence. Separate assays do not establish that metabolic suppression causes impaired bacterial clearance. |
| Stolk et al. 2020 (1) | Primary human monocytes; oxidative-burst assay. | Direct NE; in vitro. | NE 1 µM; PMA 50 ng/mL. VP 1 µM comparator. | 1 h pretreatment; luminescence recorded for 1 h after PMA. | PMA-stimulated ROS measured by a luminol assay. | NE reduced stimulated ROS output; VP did not significantly alter the response. | Oxidative-burst assay, without mitochondrial source resolution or a microbial-killing endpoint. |
| Stolk et al. 2020(1) | C57BL/6J mice with IV LPS endotoxemia. | Direct NE; in vivo. | NE 1, 3 or 5 µg/kg/min IV by osmotic pump; LPS 5 mg/kg. | LPS after 3 or 24 h of NE; assessment 90 min later (4.5 or 25.5 h total infusion). | Plasma cytokines; ex vivo neutrophil DHR oxidation and PMA-induced burst. | Cytokine balance shifted toward IL-10. Basal ROS after LPS and PMA-induced burst in non-LPS controls decreased at higher NE doses. | Endotoxemia model without live infection; effects vary by dose and assay. Mouse infusion rates cannot be directly equated with human rates. |
| Stolk et al. 2020 (1) | Mouse CLP polymicrobial sepsis. | Direct NE; in vivo. | NE 3 µg/kg/min IV; VP 0.00057 IU/kg/min or PBS comparator. | Pump placed immediately after CLP; organ/blood CFU assessed at 48 h. | Liver, spleen and blood bacterial CFU; cytokine balance. | NE increased liver and spleen CFU versus PBS. The increase in blood CFU was a non-significant trend (P = 0.06). | Functional host-defense evidence in mice. Hemodynamic or direct bacterial effects and immune mechanisms are not fully separated; metabolic mediation was not tested. |
| Stolk et al. 2020 (1) | Human endotoxemia; 30 healthy men, 10 per group. | Randomized NE exposure. | NE 0.05 µg/kg/min IV; VP 0.04 IU/min or saline; LPS 2 ng/kg IV. | 5 h infusion starting 1 h before LPS; serial measurements during the experiment. | Plasma cytokines; leukocyte counts; hemodynamics. | NE increased IL-10 and reduced IP-10. Other measured cytokines and circulating leukocyte counts were not significantly altered. | Controlled human NE exposure, but healthy endotoxemia does not reproduce septic shock. No metabolic-flux or infection-clearance endpoint. |
| Stolk et al. 2020 (1) | 195 adults with septic shock, including 129 without chronic β-blockers. | Observational cohort. | Clinically titrated NE; infusion rate recorded at blood sampling. | Within 24 h of NE initiation; median 8.8 h in the NE-only group. | Plasma TNF-α/IL-10 ratio; concurrent NE rate and β-blocker exposure. | Higher NE rate correlated with a lower TNF-α/IL-10 ratio (r = −0.38). Chronic β-blocker users had a higher ratio. | Clinical association with potential severity and treatment confounding. No direct proof of metabolic causation or immune recovery after NE reduction. |
| Estrada et al. 2016 (2) | Primary human CD8+ T cells; cytokine assays. | Direct NE; in vitro. | NE 1 or 5 µM in cytokine experiments. | Cytokine secretion assessed 24 h after TCR activation. | IFN-γ and TNF-α secretion; adrenergic receptor dependence. | NE suppressed TCR-driven cytokine secretion; β2-AR antagonism supported receptor involvement. | Activation-dependent immune effect. No septic-shock model or direct measurement of metabolic flux in these experiments. |
| Qiao et al. 2019 (3) | Murine CD8+ T cells activated with anti-CD3/CD28. | Related β-AR agonist. | Isoproterenol 10 µM; 1 µM also tested in acute flux assays. No NE intervention. | Activation for 24–48 h; glycolytic flux measured after 48 h. | GLUT1; glucose uptake; ECAR/OCR; mitochondrial mass and membrane potential. | Isoproterenol reduced glucose uptake, glycolysis and maximal respiratory capacity. Adrb2-deficient cells resisted selected effects. | Supports a β2-AR mechanism in T-cell metabolic activation. Isoproterenol exposure and a non-septic model limit direct attribution to NE in septic shock. |
| van der Heijden et al. 2020 (4) | Healthy-donor monocytes differentiated to macrophages. | Direct catecholamine training; in vitro. | NE or epinephrine 10 nM; dose-response range, 1–1000 nM.  . | 24 h exposure; 5 days of rest/differentiation; LPS restimulation on day 6. | Restimulated TNF-α; ECAR/OCR before restimulation; β-AR/cAMP dependence. | Prior exposure increased glycolysis, oxidative phosphorylation and later TNF-α responses; β-AR/cAMP inhibition prevented training. | Shows lasting, context-dependent metabolic reprogramming rather than uniform suppression. Healthy-donor model; no septic-shock or microbial-clearance endpoint. |
| van der Heijden et al. 2020 (4) | PHEO patients (n = 10) versus hypertensive controls (n = 14). | Observational. | Endogenous catecholamine excess; no assigned NE infusion dose. | Before surgery and 4 weeks after tumor removal. | Monocyte subsets; ex vivo cytokines; transcriptional and epigenetic profiles. | A pro-inflammatory monocyte phenotype and enhanced cytokine responses persisted after tumor removal. | Supports durable catecholamine-associated changes. Mixed endogenous exposure in a non-septic disease does not isolate the effect of therapeutic NE. |
| Thoppil et al. 2025 (5) | Sepsis/control ROS comparison (6 per group); control-donor PBMC stimulation (n = 7). | Observation plus direct NE in vitro. | NE 5 mM as reported; LPS 100 ng/mL or H₂O₂ 10 µM. | 16 h stimulation; then 2 h with monensin before analysis. | Total intracellular ROS by flow cytometry in CD3+ and CD14+ subsets. | Sepsis samples showed higher ROS. NE reduced stimulation-associated ROS in control PBMCs, with subset-dependent effects. | 5 mM is far above clinical circulating concentrations. Total ROS does not resolve mitochondrial versus oxidase sources; no immune-recovery or killing assay. |
| Cheng et al. 2016 (6) | LPS-tolerized human leukocytes; patient cells from sepsis with immune paralysis. | Background sepsis evidence. | N/A: NE was not the experimental intervention. | Tolerance experiments and clinical illness/recovery assessments; N/A for NE exposure. | Glycolytic and oxidative metabolism; cytokine responsiveness. | Broad metabolic defects accompanied immunoparalysis; metabolic function improved with patient recovery. | Establishes a sepsis immunometabolic phenotype. Does not identify NE as its cause or demonstrate that reducing NE restores immunity. |
| Ip et al. 2017 (7) | LPS-activated murine macrophages with IL-10 pathway manipulation. | Background mechanism. | N/A: IL-10 signaling, not NE exposure. | LPS activation and pathway-specific assays; N/A for NE exposure. | Glucose uptake/glycolysis; OXPHOS; DDIT4–mTOR activity; mitophagy and mitochondrial ROS. | IL-10 restrained glycolysis, supported oxidative metabolism and mitochondrial quality control, and reduced inflammatory mitochondrial ROS. | Supports an IL-10 metabolic mechanism. The full sequence from NE through IL-10, mTOR and mitophagy was not demonstrated in septic immune cells. |

**Abbreviations**: β-AR, β-adrenergic receptor; β2-AR, β2-adrenergic receptor; cAMP, cyclic adenosine monophosphate; CFU, colony-forming units; CLP, cecal ligation and puncture; DDIT4, DNA damage-inducible transcript 4; DHR, dihydrorhodamine; ECAR, extracellular acidification rate; GLUT1, glucose transporter 1; IFN-γ, interferon-γ; IL-10, interleukin-10; IP-10, interferon-γ-inducible protein 10; IV, intravenous; LPS, lipopolysaccharide; mTOR, mechanistic target of rapamycin; N/A, not applicable; NE, norepinephrine; OCR, oxygen consumption rate; OXPHOS, oxidative phosphorylation; PBMCs, peripheral blood mononuclear cells; PBS, phosphate-buffered saline; PHEO, pheochromocytoma; PMA, phorbol 12-myristate 13-acetate; ROS, reactive oxygen species; TCR, T-cell receptor; TNF-α, tumor necrosis factor-α; VP, vasopressin.

1.	Stolk RF, van der Pasch E, Naumann F, Schouwstra J, Bressers S, van Herwaarden AE, et al. Norepinephrine Dysregulates the Immune Response and Compromises Host Defense During Sepsis. *Am J Respir Crit Care Med* (2020) 202(6):830-42. Epub 2020/06/11. doi: 10.1164/rccm.202002-0339OC.

2.	Estrada L, Ağaç D, Farrar J. Sympathetic Neural Signaling Via the Β2-Adrenergic Receptor Suppresses T-Cell Receptor-Mediated Human and Mouse Cd8+ T-Cell Effector Function. *European Journal of Immunology* (2016) 46(8):1948-58. doi: 10.1002/eji.201646395.

3.	Qiao G, Bucsek M, Winder N, Chen M, Giridharan T, Olejniczak S, et al. Β-Adrenergic Signaling Blocks Murine Cd8+ T-Cell Metabolic Reprogramming During Activation: A Mechanism for Immunosuppression by Adrenergic Stress. *Cancer Immunology, Immunotherapy* (2019) 68(1):11-22. doi: 10.1007/s00262-018-2243-8.

4.	van der Heijden C, Groh L, Keating S. Catecholamines Induce Trained Immunity in Monocytes in Vitro and in Vivo. *Circulation Research* (2020) 127(2):269-83. doi: 10.1161/circresaha.119.315800.

5.	Thoppil J, Farrar J, Sharma D, Kirby S, Mobley A, Courtney D. Reactive Oxygen Species Elevations in Human Immune Cell Subsets During Sepsis Are Mitigated by Norepinephrine and N-Acetylcysteine. *World Journal of Critical Care Medicine* (2025) 14(4):108638. doi: 10.5492/wjccm.v14.i4.108638.

6.	Cheng S, Scicluna B, Arts R. Broad Defects in the Energy Metabolism of Leukocytes Underlie Immunoparalysis in Sepsis. *Nature Immunology* (2016) 17(4):406-13. doi: 10.1038/ni.3398.

7.	Ip W, Hoshi N, Shouval D, Snapper S, Medzhitov R. Anti-Inflammatory Effect of Il-10 Mediated by Metabolic Reprogramming of Macrophages. *Science* (2017) 356(6337):513-9. doi: 10.1126/science.aal3535.
